# RCC1L (WBSCR16) isoforms coordinate mitochondrial ribosome assembly through their interaction with GTPases

**DOI:** 10.1371/journal.pgen.1008923

**Published:** 2020-07-31

**Authors:** Aurelio Reyes, Paola Favia, Sara Vidoni, Vittoria Petruzzella, Massimo Zeviani

**Affiliations:** 1 MRC Mitochondrial Biology Unit, University of Cambridge, Cambridge, United Kingdom; 2 Dipartimento di Scienze Mediche di Base, Neuroscienze e Organi di Senso - Università degli Studi Aldo Moro, Piazza G. Cesare, Bari, Italy; The University of Western Australia, AUSTRALIA

## Abstract

Mitochondrial translation defects can be due to mutations affecting mitochondrial- or nuclear-encoded components. The number of known nuclear genes involved in mitochondrial translation has significantly increased in the past years. RCC1L (WBSCR16), a putative GDP/GTP exchange factor, has recently been described to interact with the mitochondrial large ribosomal subunit. In humans, three different RCC1L isoforms have been identified that originate from alternative splicing but share the same N-terminus, RCC1L^V1^, RCC1L^V2^ and RCC1L^V3^. All three isoforms were exclusively localized to mitochondria, interacted with its inner membrane and could associate with homopolymeric oligos to different extent. Mitochondrial immunoprecipitation experiments showed that RCC1L^V1^ and RCC1L^V3^ associated with the mitochondrial large and small ribosomal subunit, respectively, while no significant association was observed for RCC1L^V2^. Overexpression and silencing of RCC1L^V1^ or RCC1L^V3^ led to mitoribosome biogenesis defects that resulted in decreased translation. Indeed, significant changes in steady-state levels and distribution on isokinetic sucrose gradients were detected not only for mitoribosome proteins but also for GTPases, (GTPBP10, ERAL1 and C4orf14), and pseudouridylation proteins, (TRUB2, RPUSD3 and RPUSD4). All in all, our data suggest that RCC1L is essential for mitochondrial function and that the coordination of at least two isoforms is essential for proper ribosomal assembly.

## Author summary

Mitochondria are essential organelles responsible for the production of most of the energy required by the eukaryotic cells and hence their proper function is essential for cell survival. About 1,500 proteins are estimated to be present in human mitochondria but new proteins are still being reported as mitochondrial. While most proteins present in mitochondria are synthesized in the cytosol and then imported into mitochondria, 13 proteins encoded in the mitochondrial DNA are synthesized inside mitochondria by a dedicated set of proteins. RCC1L (WBSCR16) has recently been described as a protein involved in mitochondrial protein synthesis and more precisely in mitochondrial ribosome biogenesis. Three different RCC1L transcripts resulting in three different isoforms have been described; however, their role in mitochondrial translation has not been fully studied. Here we show that all three isoforms are present in mitochondria and that at least two of them play a role in mitochondrial ribosome biogenesis through the interaction with specific proteins that play a role in the assembly of either the large or the small ribosome subunit. This protein is an example of how biological processes may be more complex than anticipated as different isoforms of the same gene can have different roles in the cell.

## Introduction

Mitochondria are essential eukaryotic organelles that play important and critical roles in the cell, ranging from the biosynthesis of nucleotides, amino acids, heme, cholesterol and phospholipids to redox homeostasis, cellular waste management and apoptosis [1]. In addition, they generate ATP through oxidative phosphorylation (OxPhos) thanks to a system encompassing five multi-subunit enzymatic complexes located in the mitochondrial inner membrane. The biogenesis of these complexes is dependent upon the coordinated expression of nuclear and mitochondrial genes. While nuclear DNA codes for the majority of the structural subunits and assembly factors, mitochondrial DNA (mtDNA) still encodes 13 proteins that are essential structural subunits of complexes I, III, IV and V, two ribosomal RNAs (12S and 16S rRNA) and 22 tRNAs required for the translation of mitochondrial encoded proteins [2]. Mitochondrial translation relies on mitochondrial-specific 55S RNA-protein complexes or mitoribosomes, highly specialized in the synthesis of membrane proteins. Mitoribosomes consist of two subunits: a large 39S (mtLSU) and a small 28S (mtSSU) subunit containing mtDNA-encoded 16S and 12S rRNA, respectively and over 80 nuclear-encoded mitoribosomal proteins (MRPs) [3] [4]. In addition, mt-tRNA^Val^ or mt-tRNA^Phe^ have also been found as constituents of the mtLSU subunit, replacing bacterial and eukaryotic 5S rRNA [3] [4]. The significance of the mitoribosomes is also demonstrated by the growing number of patients with severe mitochondrial diseases associated with mutations in nuclear or mitochondrial genes coding for mitoribosomal proteins and assembly factors or RNAs [5].

Ribosome assembly is a complex series of events in which the processing and modification of rRNAs needs to be coordinated with the incorporation of MRPs. This process requires the action of many different factors such as nucleases, rRNA modifying enzymes, RNA helicases, GTPases, chaperones and maturation factors [6, 7]. GTPases are key regulators of many cellular functions such as signal transduction, cell proliferation and protein biosynthesis. GTPases can switch from an active GTP-bound conformation to an inactive GDP-bound state and an inactive intermediate apo-state. In the case of classical GTPases, state changes are mediated by the action of GTPase-activating proteins (GAPs) that promote GTP hydrolysis needed for the biological function of the protein and GDP/GTP exchange factors (GEFs) that after GTP hydrolysis replace the GDP by GTP restoring their active state [8]. However, certain ribosome-associated GTPases in bacteria such as Era, Ffh and EF-G are not reliant on GEFs for GDP/GTP exchange and they do not require GAPs for activation of GTP hydrolysis either, as this function is carried out by rRNAs instead [8].

In human mitochondria, five GTPases involved in ribosome biogenesis have been described to date: MTG1 (GTPBP7) couples mtLSU assembly with bridge formation between mtLSU and mtSSU [9] [10], MTG2 (GTPBP5) interacts with the immature mt-LSU [10], GTPBP10 is required for mtLSU late assembly [11, 12], ERAL1 stabilises 12S rRNA prior to the final maturation of the mtSSU [13] and C4orf14 binds MRPs of the mtSSU facilitating its assembly [14]. Two additional GTPases, GTPBP6 and GTPBP8, have recently been found in DDX28 pulldowns but they are still uncharacterized [12]. In addition, mitoribosomes have acquired an intrinsic GTPase activity thanks to the structural component of the mtSSU mS29, whose activity has been proposed to be linked to bridge formation between mtLSU and mtSSU, as in the case of MTG1 [4] [3]. Despite the increasing interest on mitoribosome GTPases in the last few years, very little is known about their molecular function or even if their switch from one state to another relies on GAPs/rRNA and GEFs. Indeed, the intrinsic GEF activity of mS27 on MTG1 is the only one of this kind reported so far in mammalian mitoribosomes [9].

RCC1L (WBSCR16), regulator of chromatin condensation 1 like, is a member of the RCC1 subgroup within the RCC1 superfamily of proteins characterized by the sole presence of a RCC1-like domain (RLD) that practically spans the whole length of the proteins [15]. This subgroup includes three additional members: RCC1, a GEF for the GTPase Ran [16], RCC2 (TD60), a deactivator of the GTPase Rac1 that switches its GTP for GDP [17] and DelGEF, a less characterized protein involved in regulating the GTP binding to one of the GTPases of the secretion Sec6/8 complex [18]. In mammals, RCC1L has been identified either as a component of the so called “mitochondrial pseudouridylation module”, a set of proteins involved in pseudouridylating 16S rRNA (TRUB2, RPUSD3, RPUSD4, NGRN, FASTKD2) and interacting with the mitoribosome mtLSU [19] [20] [21] [11] or as GEF for the mitochondrial fusion GTPase OPA1 [22]. However, very little attention has been paid to the fact that three different isoforms originated by alternative splicing have been identified for RCC1L, RCC1L^V1-3^, all of them sharing the same N-terminus and, therefore, all three predicted to be mitochondrial [20].

In this study, we show that all three isoforms of RCC1L are mitochondrial proteins, the most and least abundant being RCC1L^V1^ and RCC1L^V3^, respectively. They are peripherally associated to the mitochondrial inner membrane, showing RCC1L^V2^ the strongest association. Isokinetic sucrose gradients and immunoprecipitation experiments showed that RCC1L^V1^ and RCC1L^V3^ associated with mt-LSU and mt-SSU, respectively while no significant association was observed for RCC1L^V2^. Both overexpression and silencing of RCC1L^V1^ or RCC1L^V3^ led to mitoribosome biogenesis defects that resulted in decreased translation, while RCC1L^V2^ overexpression increased translation. Changes in the steady-state level and distribution of mitoribosome GTPases on sucrose gradients were also detected, suggesting a role of RCC1L isoforms in their activation/deactivation. Altered RCC1L^V1^ expression changed GTPBP10 levels and distribution in mtLSU along with components of the pseudouridylation module, TRUB2, RPUSD3 and RPUSD4, and it was greatly enriched in RCC1LV^1^ pulldowns. RCC1L^V3^ altered expression greatly increased ERAL1, overall and in fractions with mtSSU subcomplexes, and C4orf14 in mtSSU. Moreover, a fraction of each isoform associated with the other isoforms independently of DNA or RNA. Furthermore, RCC1L^V2^ displayed DNA binding properties, while the other two had a weak affinity for poly-U but none of them was able to bind poly (A) templates. Our data suggests that RCC1L^V1^ and RCC1L^V3^ have a role in the assembly of mtLSU and mtSSU, respectively and points towards mitoribosome GTPases GTPBP10, ERAL1 and possibly C4orf14 as likely putative targets.

## Results

### RCC1L isoforms localise to the mitochondrial matrix

**Fig 1:**
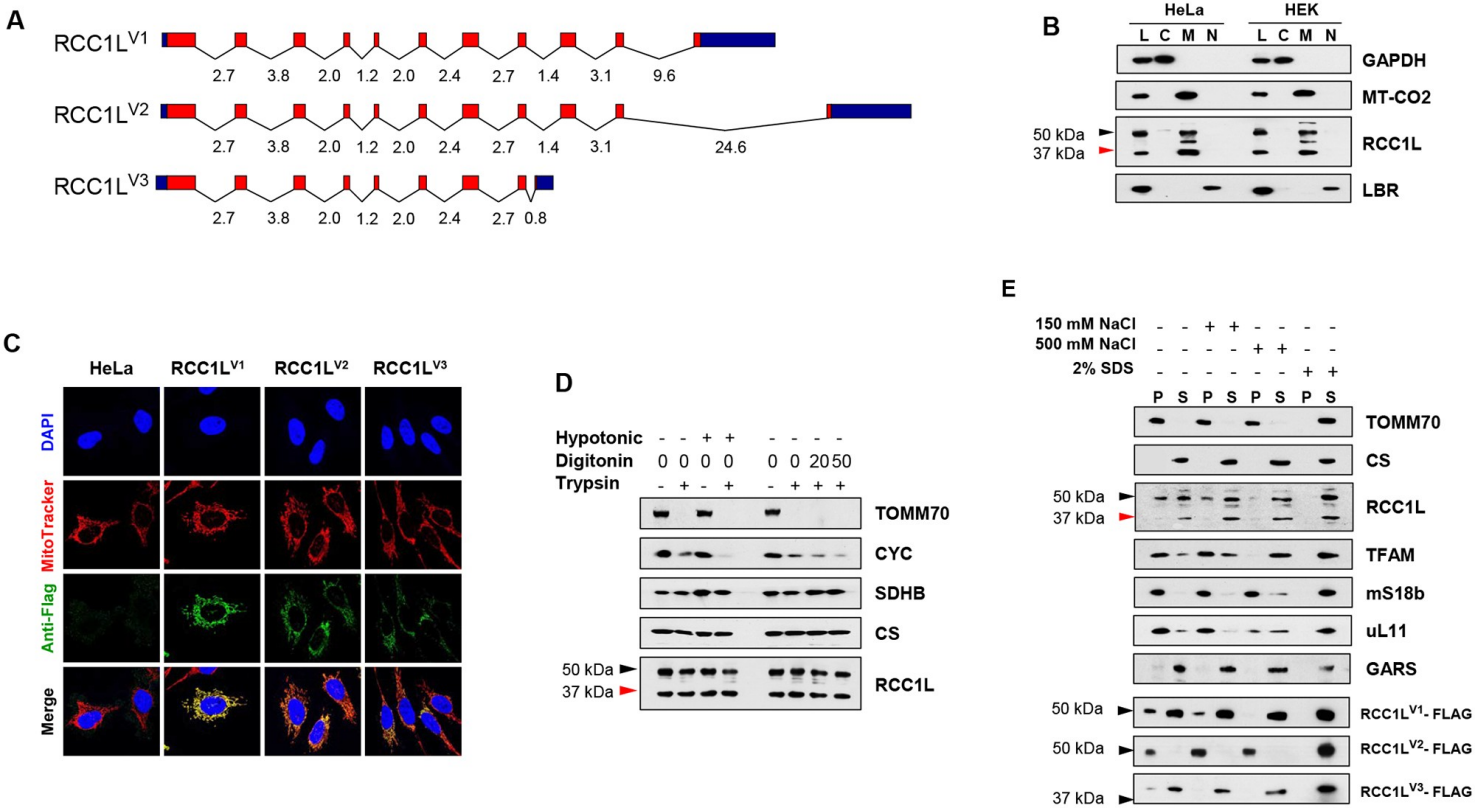
RCC1L isoforms are all mitochondrially localised. (**A**) Schematic representation of the three RCC1L isoforms generated by alternative splicing showing the UTRs (bue), exons (red) and introns with their lengths in kilobases. (**B**) Immunoblot analysis of RCC1L isoforms in parental HeLa and HEK whole cell lysate (L), cytosolic (C), mitochondrial (M) and nuclear (N) fractions. Antibodies against well-known fraction-specific proteins were used as control: GAPDH, MT-CO2 and LBR for the cytosolic, mitochondrial and nuclear fractions, respectively. (**C**) Intra-cellular localisation of RCC1L isoforms by immunofluorescence. Parental and transfected HeLa cells expressing a STREP2-FLAG-tagged version of each isoform were stained with DAPI for the nucleus, MitoTracker Red for mitochondria and anti-FLAG antibody followed by Alexa 488 conjugated secondary antibody for the overexpressed RCC1L proteins. Co-localisation of MitoTracker and RCC1L-specific green signal appears yellow to orange, depending on the abundance, in the merged images. (**D**) Sub-mitochondrial localisation of RCC1L isoforms. Purified mitochondria from parental HEK cells were incubated in isotonic, hypotonic buffer or isotonic buffer with increasing concentrations of digitonin (0–50 10 mg/ml) before treatment with trypsin (100 μg/ml for 30 min). Sub-mitochondrial localisation was determined as mitochondrial inner membrane or matrix based on the resistance to trypsin in the different conditions and by comparison to control proteins localised in the mitochondrial outer membrane (TOMM70), inter-membrane space (CYC), inner membrane (SDHB) and matrix (CS) by immunoblotting. (**E**) Mitochondrial membrane association of RCC1L isoforms. Purified mitochondria from parental HEK cells were sonicated and, after high speed centrifugation, membrane fractions were recovered from the pellet (P), leaving non-membrane associated proteins in the supernatant (S) and were analysed by immunoblotting. Recovery was performed with three different NaCl concentrations and under full solubilisation conditions (2% SDS). A mitochondrial transmembrane protein and a soluble matrix protein, TOMM70 and CS, respectively, were used as controls. Additional proteins such as the nucleoid protein TFAM, mitochondrial ribosomal proteins mS18b and uL11 and tRNA synthetase GARS were also used as controls. STREP2-FLAG-tagged overexpressed RCC1L isoforms were also analysed under the same conditions. In the immunoblots of endogenous RCC1L, the 50 kDa band (black arrowhead) contains isoforms RCC1L^V1^ and RCC1L^V2^, while the 37 kDa band (red arrowhead) corresponds to isoform RCC1L^V3^.

In humans, three different isoforms have been reported in Ensemble for RCC1L (Fig 1A). RCC1L^V1^ is 464 amino acids long and is considered the principal isoform. RCC1L^V2^ is 454 amino acids long and results from alternative splicing of the last exon. The difference between these two isoforms resides exclusively in the C-terminal 25 amino acids of the protein. RCC1L^V3^ is much shorter, 358 amino acids, since alternative splicing of exon 9 creates an early stop codon and a shorter transcript. A search for the presence of RCC1L isoforms in Ensemble revealed that most mammalian species, representing nine different orders, only presented one isoform, RCC1L^V1^ (S1 Fig). Two isoforms, RCC1L^V1^ and RCC1L^V2^, were found in prosimian primates, while all three isoforms were present in anthropoid primates (S1 Fig). These results suggest that isoform RCC1L^V1^ was the original and single isoform present in mammalian species and that isoforms RCC1L^V2^ and RCC1L^V3^ appeared later on during primate evolution.

In silico analysis of human RCC1L isoforms predicted that all three isoforms would be targeted to mitochondria with high scores and the presence of a cleavable mitochondrial targeting sequence (MTS) (S2 Fig). Since all three isoforms share the same N-terminus, no difference in mitochondrial localization was predicted. Indeed, cellular fractionation experiments in HeLa and HEK cells revealed the present of two RCC1L bands in purified mitochondria: one band of about 50 kDa comprising isoforms RCC1L^V1^ and RCC1L^V2^, that could not be separated as the difference between them is about 1 kDa, and another band at about 37 kDa corresponding to isoform RCC1LV^3^ (Fig 1B). A much weaker band and of slightly higher molecular weight was also detected in the cytosolic fractions and cell lysates. This band may correspond to one of the precursors of the proteins still containing the MTS attached based on the size and cellular localization. In some occasions, an additional band of about 45 kDa, corresponding to a degradation product, was detected in purified mitochondria that were treated with trypsin (Fig 1B) but not in untreated mitochondria nor in whole cell lysates (Fig 1B and 1D). In addition, we overexpressed FLAG-tagged versions of each isoform in HeLa cells and analysed their localization by immunofluorescence. While all three isoforms were found to colocalise with mitochondria (Fig 1C), a markedly difference in the level of expression was also observed, which may explain the differential effect they have on other proteins, i.e., the higher the level of overexpression the more evident the effect (see below). Furthermore, deletion of the predicted N-terminal MTS (37 amino acids) completely abolished mitochondrial localization of the protein and led to protein degradation (S3 Fig).

In order to get further insight into the submitochondrial localisation of RCC1L, trypsin protection experiments were carried out in HEK cells. Mitochondrial outer membrane (TOMM70) and intermembrane space (CYC) control proteins were significantly degraded by trypsin in hypotonic- or digitonin-treated samples (Fig 1D). By contrast, no significant decrease in either the RCC1L 50 nor the 37 kDa band was detected after treatment with trypsin. Same results were also observed in mitochondrial inner membrane (SDHB) and matrix (CS) protein controls (Fig 1D). In order to differentiate between these two mitochondrial localisations, membrane (P) and soluble (S) fractions from HEK mitochondria were separated in the presence of increasing salt concentration (Fig 1E). RCC1L bands were detected both in the soluble and membrane fractions, however their ratio shifted towards the soluble fraction as salt concentration increased, pointing to a mitochondrial matrix localization with inner membrane interaction. As expected, similar behaviour was found in proteins constituents of the mitochondrial nucleoids and mitoribosomes, both inner membrane-interacting structures. Additional information was obtained from the analysis of overexpressed FLAG-tagged versions of each isoform in HEK cells. While FLAG-tagged isoforms RCC1L^V1^ and RCC1L^V3^ confirmed the distribution observed in the endogenous protein, FLAG-tagged isoform RCC1L^V2^ was always found in the membrane fraction, unless solubilised with 2% SDS, indicating a tighter interaction with mitochondrial inner membrane structures (Fig 1E).

### RCC1L overexpression alters mitochondrial protein and RNA levels

**Fig 2:**
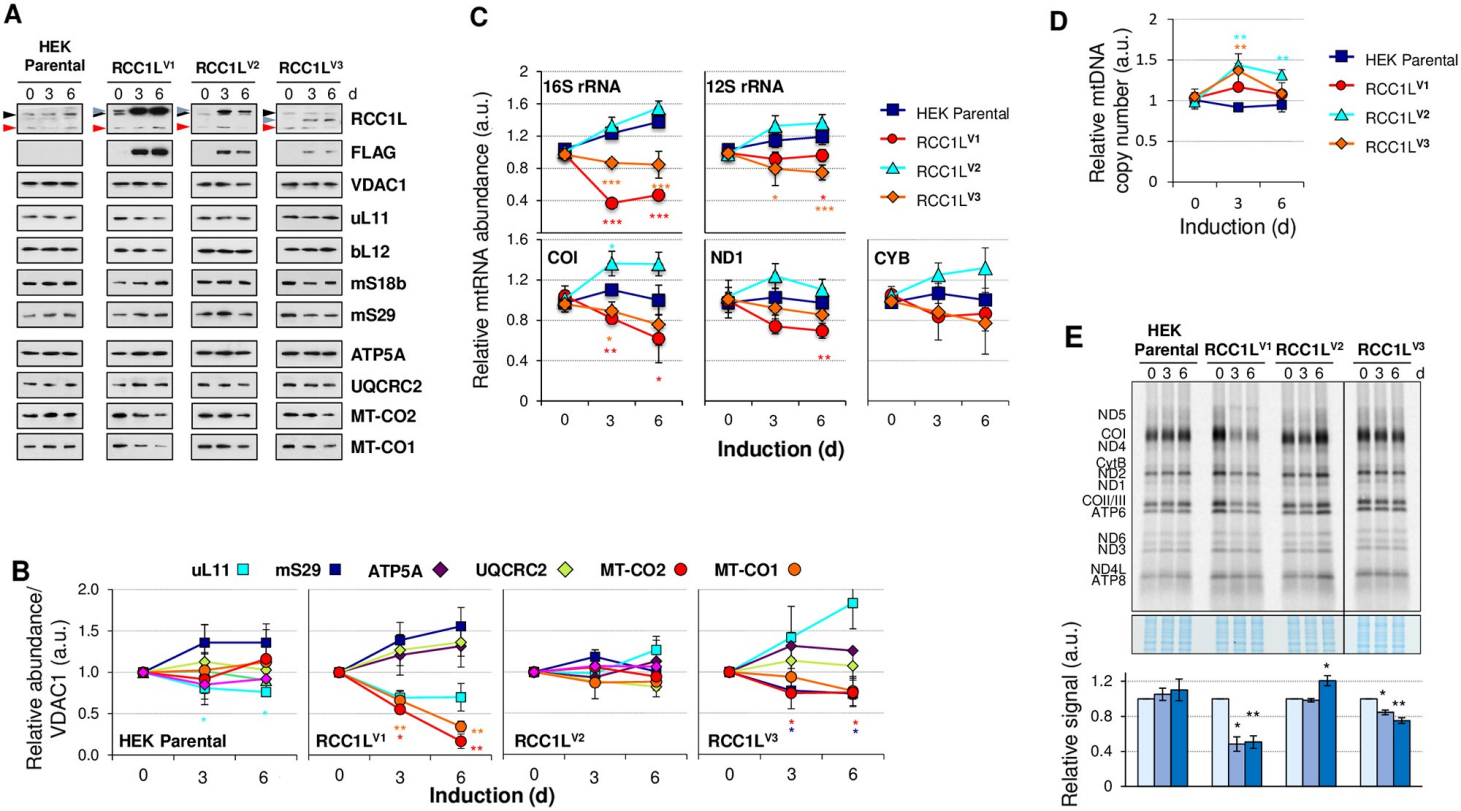
Overexpression of RCC1L isoforms alters the expression of mitochondrial proteins. (**A**) Immunoblot of the STREP2-FLAG-tagged RCC1L expression in purified mitochondria from HEK after induction with 10 ng/ml doxycycline for 0, 3 and 6 days using both RCC1L and FLAG primary antibodies. In the immunoblots of endogenous RCC1L, the 50 kDa band (black arrowhead) contains isoforms RCC1L^V1^ and RCC1L^V2^, while the 37 kDa band (red arrowhead) corresponds to isoform RCC1L^V3^. The STREP2-FLAG-tagged RCC1L proteins (RCC1L^V1^, RCC1L^V2^ and RCC1L^V3^) are also marked (grey arrowheads) in each case. Steady-state levels of mtLSU (uL11, bL12), mtSSU (mS18b, mS29) and components of the respiratory chain (ATPA5, UQCRC2, MT-CO2, MT-CO1) were also verified. VDAC1 was used as mitochondrial loading control. (**B**) Quantification of steady state levels of selected mitochondrial proteins from panel (A) based on densitometric analysis of the immunoblots and normalised to loading control VDAC1. (**C**) Quantitative PCR (qPCR) analysis of the steady state levels of representative mtDNA-encoded mRNAs (MT-CO1, MT-ND1, MT-CYB) and rRNAs (16S and 12S) normalised to GAPDH mRNA levels in parental and RCC1L overexpressing cells after 0, 3 and 6 days of induction. (**D**) qPCR analysis of mitochondrial DNA copy number in parental and RCC1L overexpressing cells after 0, 3 and 6 days of induction. Data represent the average of MT-CO1, MT-CYB and 12S rRNA normalised to APP. (**E**) [^35^S]-methionine *de novo* synthesis of mitochondrially encoded proteins in parental and RCC1L overexpressing cells after 0, 3 and 6 days of induction. Newly synthesized proteins were visualized after exposure of the dried gel to phosphorscreens (above) and signal intensity was quantified with ImageQuant software. Coomassie stain signal was used as loading control for normalisation (below). Samples corresponding to overexpression of RCC1L^V3^ were run on a separate gel. In panels (B-E), data represent mean ± SD from three independent experiments. t-test: *P < 0.05, **P < 0.01, ***P < 0.001 with colours to match the sample. See S2 Table for quantitative data in this figure.

RCC1L was first identified in a screening of genes required for OxPhos [20], therefore our first approach was to study the effect that overexpression of the different RCC1L isoforms may have on mitochondrial proteins and RNAs (Fig 2). Culture of parental HEK cells in media containing doxycycline for three or six days marginally altered the levels of mS29 and uL11, without affecting the steady state levels of Oxphos proteins ATP5A, UQCRC2, MT-CO1 or MT-CO2 (Fig 2A and 2B). Overexpression of RCC1L isoforms produced very different profiles. On one hand, full induction of the transgene expression resulted in very different levels of tagged protein for each isoform, being RCC1L^V1^ four and 15 times more expressed than RCC1L^V2^ and RCC1L^V3^, respectively (Fig 2A). These results were in line with the confocal microscopy data obtained in HeLa cells (Fig 1C). On the other hand, overexpression of each isoform resulted in an isoform specific pattern of mitoribosome and OxPhos proteins (Fig 2A and 2B). Overexpression of RCC1L^V1^ led to a decrease of mtLSU component uL11 and an even more pronounced decrease in mitochondrially encoded proteins, MT-CO1 and MT-CO2, accompanied by a moderate or slight increase of mtSSU protein mS29 and OxPhos proteins ATP5A and UQCRC2 (Fig 2A and 2B). No change in protein levels was observed in HEK cells overexpressing RCC1L^V2^. Actually, induction with doxycycline in RCC1L^V2^ resulted in less changes than in parental HEK cells in some cases (Fig 2A and 2B). Despite the levels of overexpressed RCC1L^V3^ being lower than RCC1L^V1^, we were able to detect a decreasing trend in mtSSU protein mS29, MT-CO1 and MT-CO2 along with an increase in mtLSU component uL11 but no change in OxPhos proteins ATP5A and UQCRC2 (Fig 2A and 2B).

Since we observed a decrease in mitochondrially encoded proteins MT-CO1 and MT-CO2, we also checked mitochondrial RNA levels (Fig 2C). Parental HEK cells and those overexpressing isoform RCC1L^V2^ had increased RNA levels for both rRNAs and mRNAs, being more pronounced in the RCC1L^V2^ expressing cells (Fig 2C). However, cells overexpressing either isoform RCC1L^V1^ or RCC1L^V3^ displayed a decrease in RNA levels, being more prominent in the former isoform, most likely due to the higher level of protein overexpression. The most affected transcripts in RCC1L^V1^ overexpression were 16S rRNA followed by MT-CO1 mRNA, while RCC1L^V3^ overexpression reduced the levels of MT-CO1 transcript and to a lesser degree 12S rRNA and MT-CYB mRNAs (Fig 2C). These changes in transcript levels were not due to changes in mitochondrial DNA levels since all three cell lines overexpressing RCC1L isoforms displayed mild increased mitochondrial DNA levels at three days of induction to a similar extent and values similar to parental cell line at six days, except in the case of RCC1L^V2^ where values remained slightly higher also after six days of induction (Fig 2D).

The observed effects of overexpression on mitochondrial rRNAs, specially on 16S rRNA, prompted us to examine the mitochondrial translation capacity of cells overexpressing each RCC1L isoform. Mitochondrial protein synthesis rates in parental HEK cells were not significantly changed by the addition of doxycycline for three or six days (Fig 2E). However, overexpression of RCC1L^V1^ significantly decreased *de novo* mitochondrial protein synthesis by 50% compared to non-induced cells at both three and six days of induction (Fig 2E). A significant but much smaller decrease in mitochondrial translation was also observed when overexpressing RCC1L^V3^, 15% and 25% at three and six days of induction, respectively. By contrast, overexpression of RCC1L^V2^ did not alter mitochondrial protein synthesis rate after three days of induction but significantly promoted protein synthesis after six days of induction (Fig 2E). Mitochondrial translation results suggested a translation defect, most likely due to alterations in mitoribosome assembly, that could easily explain the mitochondrial rRNA and protein level changes observed in these cells (Fig 2A–2C).

### RCC1LV^1^ and RCC1LV^3^ isoforms interact with different mitoribosome subunits

**Fig 3:**
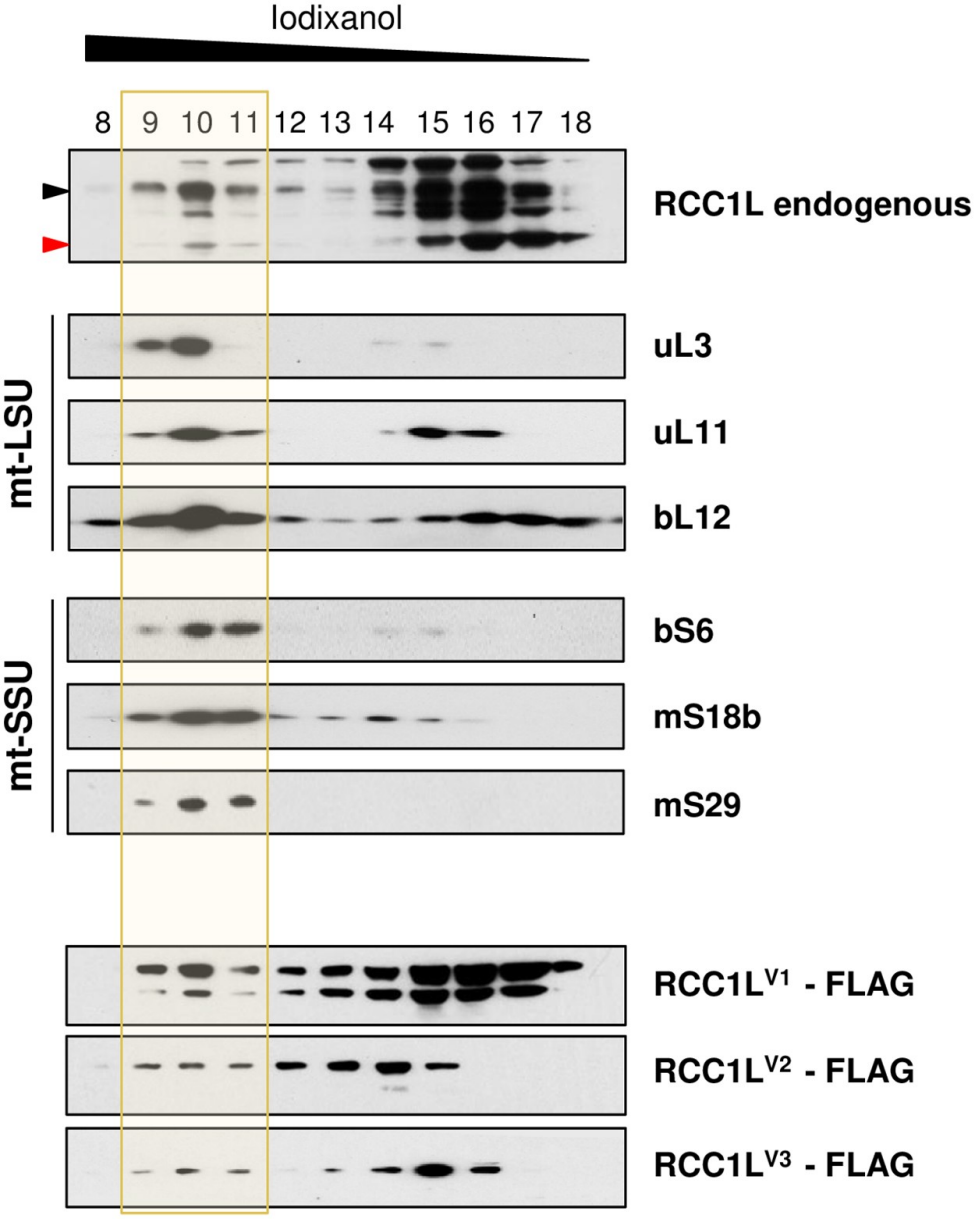
RCC1L isoforms and mitochondrial ribosomal subunits in iodixanol gradients. Mitochondrial RCC1L and ribosome profiles in parental and RCC1L overexpressing cells after induction with 10ng/ml doxycycline for 3 days. Mitochondrial lysates were separated on a 20–45% (v:v) self-forming iodixanol gradient and fractions were analysed by immunoblotting. In the immunoblots of endogenous RCC1L, the 50 kDa band (black arrowhead) contains isoforms RCC1L^V1^ and RCC1L^V2^, while the 37 kDa band (red arrowhead) corresponds to isoform RCC1L^V3^. Antibodies against structural components of mtLSU (uL3, uL11 and bL12) and mtSSU (bS6, mS18b and mS29) were used for immunodetection. Transparent yellow colour mark the fractions where mitochondrial ribosomal subunits are present (fractions 9–11) whereas non-assembled subunit peaks are left unmarked (fractions 12–18). Equal volume of each fraction was loaded for all cell lines.

To gain insight into the possible role of RCC1L in mitochondrial translation and mitoribosome assembly, we explored the possible association of different RCC1L isoforms with mitoribosome subunits by iodixanol and sucrose gradient sedimentation analysis of mitochondrial extracts. Iodixanol gradients performed in parental HEK cells showed that a significant amount of endogenous RCC1L cofractionated with both mtLSU and mtSSU (Fig 3). Fraction 10 was enriched in mtLSU proteins while fractions 10 and 11 contained similar levels of mtSSU proteins. Also endogenous 50 kDa RCC1L was found significantly enriched in fraction 10 and present in fractions 9 and 11 in a fashion similar to mtLSU markers (uL3, uL11 and bL12) with the free protein in fractions 14–17. Endogenous 37 kDa RCC1L was higher in fraction 10 than in fraction 11 and this pattern was also mirrored by mtSSU markers (bS6, mS18b and mS29), albeit with a less pronounced difference between fractions. Free 37 kDa RCC1L was present in fractions 15–18 (Fig 3). When iodixanol gradients were performed on HEK cells overexpressing FLAG-tagged version of each isoform, a consistent pattern with the endogenous protein was observed. Overexpressed RCC1L^V1^ displayed an overall pattern that matched well that of the endogenous 50 kDa RCC1L, including a higher abundance in fraction 10 than in fractions 9 and 11. A different result was obtained with the overexpressed RCC1L^V2^. This protein seemed to trail from the free protein in fractions 12–15 to fractions 9–11. Finally, overexpressed RCC1L^V3^ was present in fractions 10 and 11 in similar ratios to the endogenous 37 kDa RCC1L, while the free protein was present in lower fractions than the latter (fractions 13–16 compared to 15–18) but closer to some mtSSU free protein fractions (bS6 fractions 14–15 or mS18b 12–15) (Fig 3).

**Fig 4:**
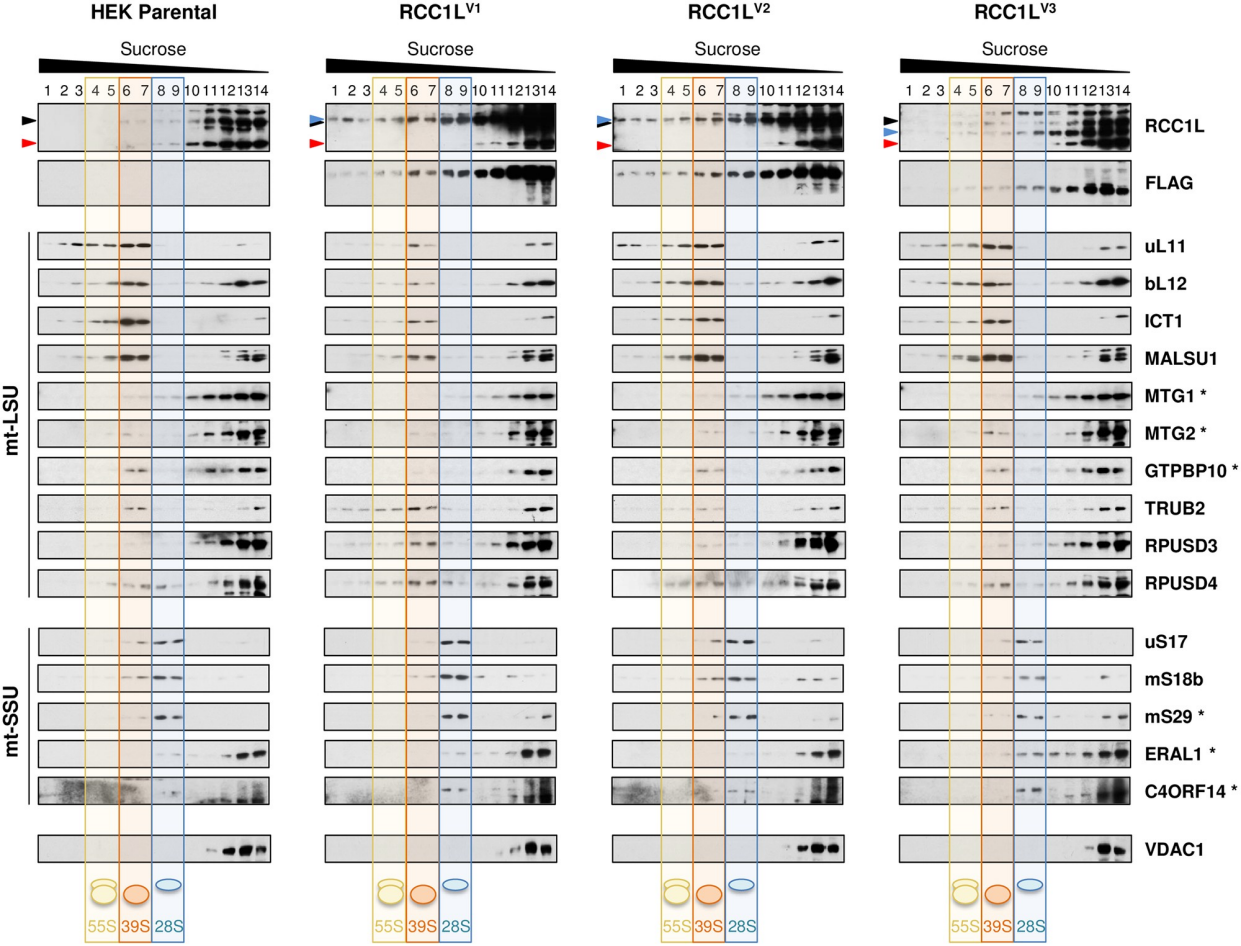
RCC1L isoforms and mitochondrial ribosomal subunits in isokinetic sucrose gradients. Mitochondrial ribosome profile in parental and RCC1L overexpressing cells after induction with 10ng/ml doxycycline for 3 days. Equal amounts of mitochondrial lysates from each of the four cell lines were separated on a 10–30% (v:v) isokinetic sucrose gradient and fractions were analysed by immunoblotting. In the immunoblots of endogenous RCC1L, the 50 kDa band (black arrowhead) contains isoforms RCC1L^V1^ and RCC1L^V2^, while the 37 kDa band (red arrowhead) corresponds to isoform RCC1L^V3^. The STREP2-FLAG-tagged RCC1L proteins (RCC1L^V1^, RCC1L^V2^ and RCC1L^V3^) are also marked (grey arrowheads) in each case. Antibodies against structural components (uL11, bL12, ICT1) and assembly factors (MALSU1) of the mtLSU along with GTPases (MTG1, MTG2, GTPBP10) and components of the pseudouridylation module (TRUB2, RPUSD3 and RPUSD4) involved in mtLSU biogenesis were used for immunodetection of proteins of interest. In the case of mtSSU analysis, immunoblot analysis was performed using antibodies against structural components (uS17, mS18b, mS29) and related GTPases (ERAL1, C4ORF14). Immunoblots for additional mtLSU and mtSSU proteins are shown in S4 Fig. Transparent blue, orange and yellow colours mark the fractions where the 28S mtSSU (fractions 8–9), 39S mtLSU (fractions 6–7) and 55S monosome (fractions 4–5) peak, respectively whereas non-assembled subunit peaks are left unmarked (fractions 10–14). Equal volume of each fraction was loaded for all cell lines.

In the case of sucrose gradients, the cosedimentation of RCC1L isoforms with mtLSU and mtSSU markers is less evident. In parental HEK cell line, some endogenous 50 kDa RCC1L was found in fractions containing mtLSU markers (uL11, bL12 and ICT1) and assembly factor (MALSU1), as in fractions 6 and 7 (Fig 4, S4 and S5 Figs). Similarly, a fraction of the GTPases involved in mtLSU assembly and pseudouridylation module components were also detected in these fractions, being GTPBP10, TRUB2 and RPUSD4 the most abundant ones. Moreover, some endogenous 37 kDa RCC1L was present in fractions with mtSSU markers (uS17, mS18b and mS29) and GTPases ERAL1 and C4ORF14, as in fractions 8 and 9 (Fig 4, S4 and S5 Figs). In order to get further insight, sucrose gradients were performed on HEK cells overexpressing FLAG-tagged version of each isoform. The overexpression of RCC1L^V1^ showed an small increase of this isoform in fraction 6 compared to nearby fractions, as also detected in other mtLSU markers (Fig 4, S4 and S5 Figs). In addition, we observed a dramatic decrease in the abundance of mtLSU markers and assembly factors accompanied by a significant increase in all three pseudouridylation module components compared to parental HEK, specially in fractions 6 and 7 (Fig 4, S5 and S6 Figs). A slight increase of mtSSU markers and C4ORF14 levels in fractions 8 and 9 compared to HEK parental cells were also detected. Overexpressed RCC1L^V2^ distributed along the gradient without showing any significant accumulation in any fraction (Fig 4, S4 and S5 Figs). However, structural components and some associated factors showed minor random changes, e.g., ICT1, TRUB2 and ERAL1 were decreased but MALSU1, RPUSD3 and uS17 were increased compared to control in either fractions 6–7 or 8–9 (Fig 4, S5 and S6 Figs). The level of overexpressed RCC1L^V3^ isoform was again lower than the levels of the other two isoforms, in agreement with the results shown above (Figs 1C and 2A). In spite of this, overexpressed RCC1LV^3^ displayed a small shoulder in frations 9 and 10, slightly earlier than the mtSSU markers, peaking in fractions 8 and 9 (Fig 4, S4 and S5 Figs). While mtSSU markers did not change their sedimentation profile, they were slightly decreased. By contrast, GTPases EARL1 and C4ORF14 were increased, with the former showing a distribution that to a great extend shadowed the tagged protein. Furthermore, endogenous 50 kDa RCC1L was found slightly increased, mainly in fraction 6, compared to nearby fractions, and as did several mtLSU markers, assembly factors and pseudouridylation module components (Fig 4, S5 and S6 Figs).

**Fig 5:**
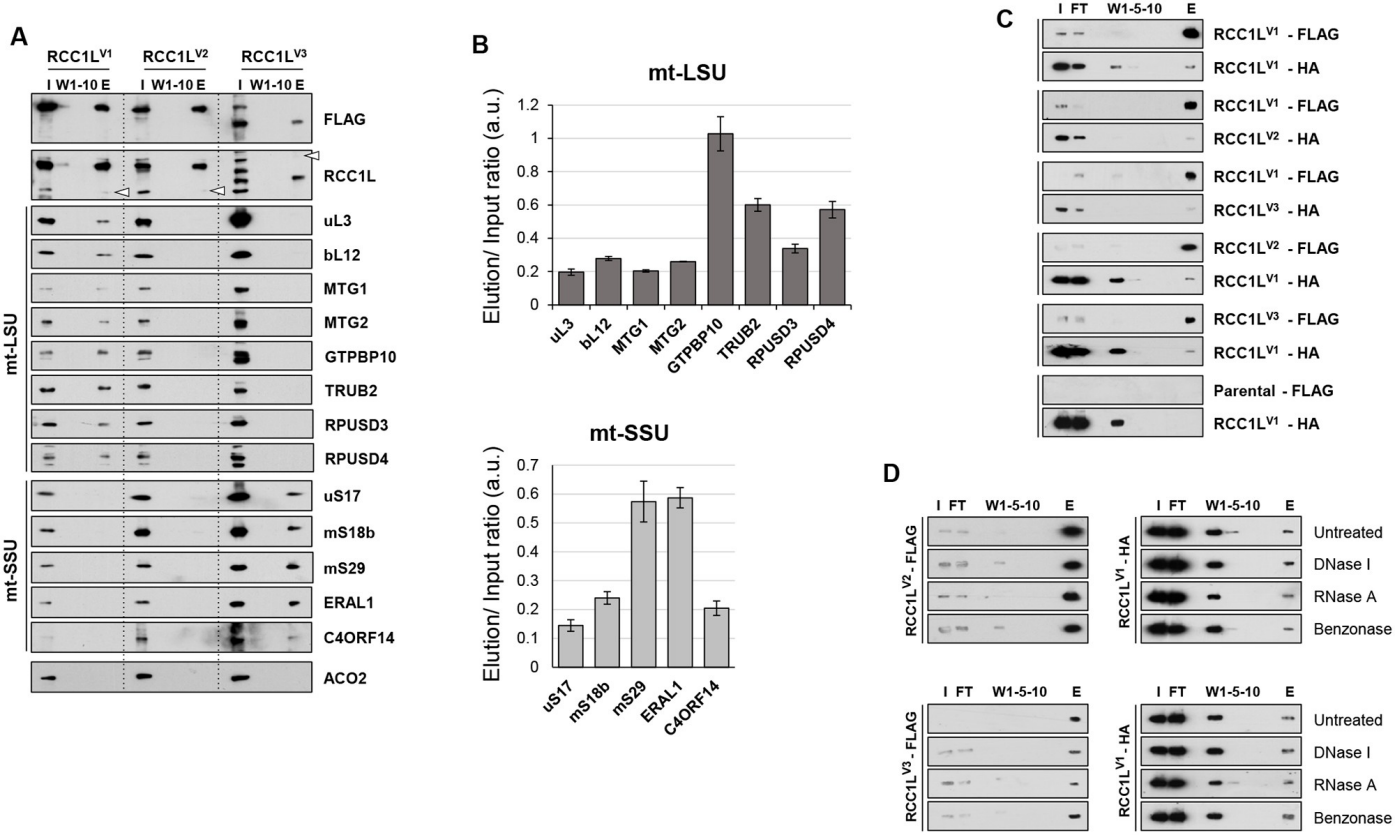
RCC1L strongest interactors are mitoribosome biogenesis factors. (**A**) Co-immunoprecipitation of STREP2-FLAG-tagged RCC1L isoforms from purified mitochondria after induction of HEK cells with 3–10 ng/ml doxycycline for 3–4 days. In the immunoblots of endogenous RCC1L, the endogenous isoforms (37 or 50 kDa band) are marked (empty arrowhead) in the elution fractions. Immunoblots for mitochondrial ribosomal proteins and biogenesis factors are presented. ACO2 was used as mitochondrial negative control for unspecific binding. Additional immunoblots and longer exposures are shown in S5A and S5B Fig, respectively. (**B**) Quantification of pull-down enrichment of mtLSU and mtSSU proteins from panel (A) based on densitometric analysis of the elution signal and normalised to its input loading. Data represent mean ± SD from two independent experiments. (**C**) Co-immunoprecipitation of STREP2-FLAG-tagged and HA-tagged RCC1L isoforms from HEK purified mitochondria. Cells overexpressing STREP2-FLAG-tagged RCC1L^V1^ were transiently overexpressing HA-tagged version of each of the three isoforms: RCC1L^V1^, RCC1L^V2^ and RCC1L^V3^. Alternatively, cells overexpressing STREP2-FLAG-tagged RCC1L^V2^ or RCC1L^V3^ were transiently overexpressing HA-tagged RCC1L^V1^. Parental HEK cells not expressing any STREP2-FLAG-tagged protein but transiently overexpressing HA-tagged RCC1L^V1^ were used as negative control for unspecific pull down. (**D**) Interaction between RCC1L isoforms is independent of nucleic acids. Cells overexpressing STREP2-FLAG-tagged RCC1L^V2^ or RCC1L^V3^ were transiently overexpressing HA-tagged RCC1L^V1^. Mitochondrial lysates were split into four identical columns and before elution they were left untreated or treated with DNase I (20U/ml), RNase A (40 μg/ml) or Benzonase (12.5 U/ μl) for 1 h at 4°C. The effectiveness of the treatments was verified by measuring the amount of DNA or RNA present in the elution by qPCR (S5C Fig). Input (I) and flow through (FT) 3%, washes (W1-5-10) and elution (E) 8%. See S3 Table for quantitative data in this figure.

Co-immunoprecipitation experiments using overexpressed FLAG-tagged versions of the proteins were performed in order to assess the possible interaction of RCC1L isoforms with the mtLSU and mtSSU. When RCC1L^V1^ was used as a bait, we were able to detect in the elution mtLSU MRPs as well as GTPases associated with the assembly of the mtLSU and components of the pseudouridylation module (Fig 5A, S7A and S7B Fig). GTPBP10 was the most enriched protein in the elution followed by pseudouridylation proteins TRUB2 and RPUSD4, about five and three times more enriched than the rest of the proteins, respectively (Fig 5B and S7B Fig). Only traces of some mtSSU MRPs were detected in longer exposures of the blots (S7B Fig), supporting the interaction of RCC1L^V1^ with the mtLSU and in agreement with the data from the iodixanol and sucrose gradients (Figs 3 and 4). By contrast, when RCC1L^V3^ was used as a bait, we detected mtSSU MPRs in the elution and GTPases associated with this subunit, but only traces of components of the mtLSU in the longer exposures (Fig 5A, S7A and S7B Fig). The two most enriched proteins in the elution were two GTPases, mS29, which is also a structural subunit of mtSSU that has retained GTPase activity, and ERAL1 (Fig 5B). Both of them were found to be about three times more abundant than the rest of the proteins found in the elution. The results on ERAL1 corroborated those obtained with the sucrose gradients from cells overexpressing RCC1L^V3^ (Fig 4). However, the increase detected for C4ORF14 in the sucrose gradients was not accompanied by a high enrichment in the co- immunoprecipitation, suggesting a different regulation by the GTPase or a lower affinity for mitoribosomes in the absence of GTP in the elution buffer [14]. A completely different picture was obtained when RCC1L^V2^ was used as a bait (Fig 5A S7A and S7B Fig). No significant amount of any protein, neither structural nor GTPase or from the pseudouridylation module, was retrieved in the elution, albeit small traces of some of them were detected in the longer exposures (Fig 5A, S7A and S7B Fig). These results, along with those from the sucrose gradients (Fig 4) suggested that RCC1L^V2^ was not directly interacting with any structural subunit or assembly factor of the mitoribosome, despite being 95% identical to RCC1L^V1^ and with only 25 amino acid difference at the C-terminus.

Additional unexpected results were also obtained from the co-immunoprecipitation experiments. i.e., RCC1L blots revealed that endogenous 37 kDa RCC1L was found in the elution of both RCC1L^V1^ and RCC1L^V2^ while 50 kDa RCC1L was found in the elution of RCC1L^V2^ (Fig 5A and S7B Fig), suggesting interaction among the different isoforms. In order to corroborate these results and get isoform specific information, we overexpressed two of the isoforms at a time, each with a different tag, FLAG or HA. When we used RCC1L^V1^-FLAG as bait, we were able to detect all three HA-tagged isoforms, RCC1L^V1^, RCC1L^V2^ and RCC1L^V3^, with a relative abundance that was proportional to the level of overexpression for each isoform (Fig 5C). Similarly, when we used either RCC1L^V2^-FLAG or RCC1L^V3^-FLAG, we could detect RCC1L^V1^-HA, but that was not the case if parental HEK cells not expressing any FLAG-tagged protein were used (Fig 5C). Treatment of the columns with DNase I, RNase A or Benzonase prior to elution, did not significantly changed the yield of HA-tagged proteins in the elution despite effectively degrading DNA, RNA or both (Fig 5C and S7C Fig). Hence, interaction between the different isoforms was not mediated by nucleic acids but most likely due to protein-protein interaction. However, it is noteworthy to mention that the amount of HA-tagged protein pulled down with the FLAG-tagged protein only represented a small fraction of the input and therefore, RCC1L complexes may not be very abundant in physiological conditions.

### Silencing of RCC1L promotes mitochondrial translation defects

**Fig 6:**
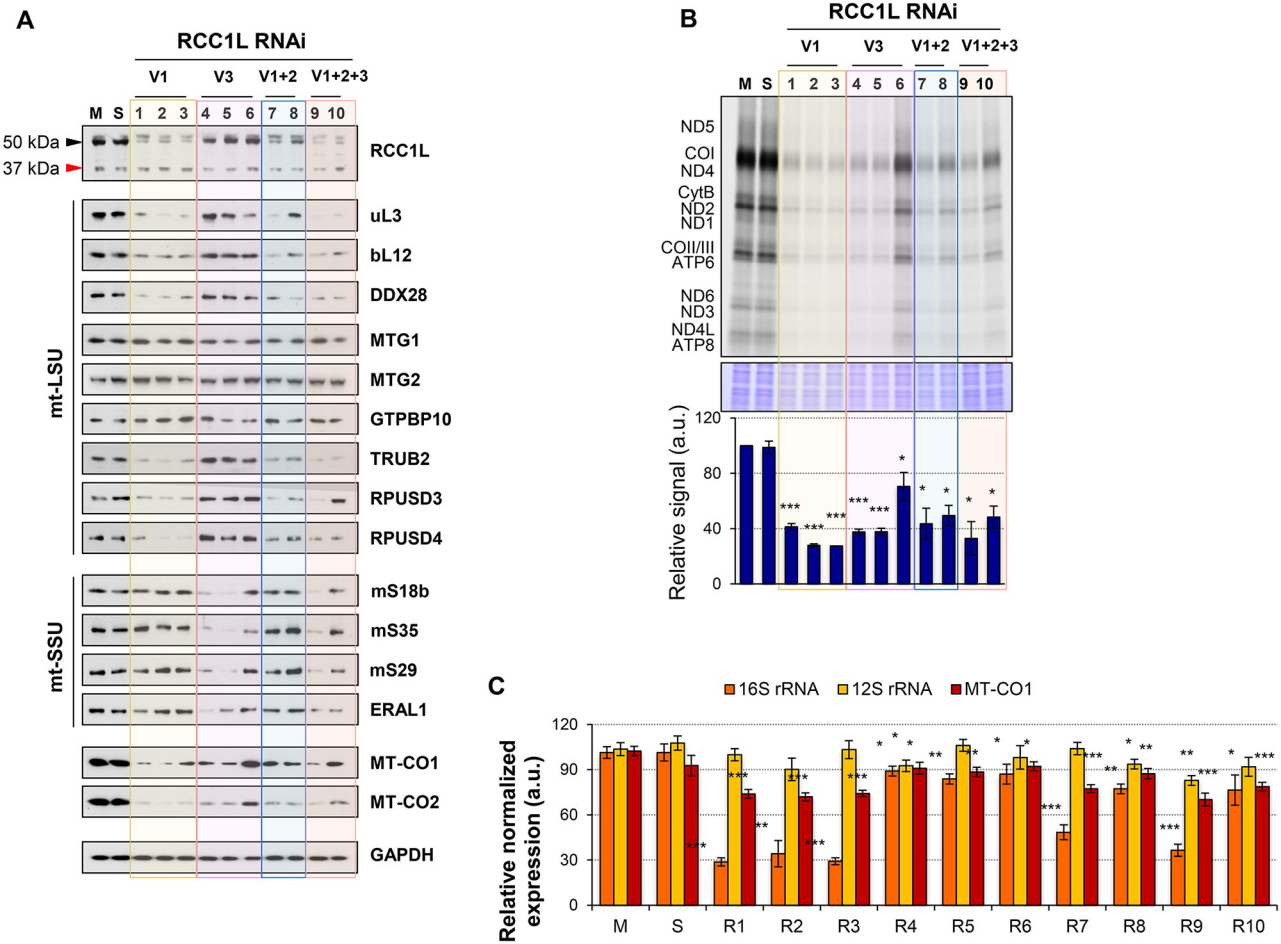
Silencing of RCC1L isoforms alter the levels of mitochondrial ribosomal proteins and biogenesis factors resulting in decreased mitochondrial translation. (**A**) Silencing of RCC1L^V1^ (V1, RNAi 1–3) and RCC1L^V3^ (V3, RNAi 4–6) isoforms and combined silencing of RCC1L^V1^ and RCC1L^V3^ (V1+2, RNAi 7–8) and all three isoforms (V1+2+3, RNAi, 9–10) in HEK cells. Mock (M) and AllStars dsRNA (S) transfected cells were used as negative controls. In the immunoblots of endogenous RCC1L, the 50 kDa band (black arrowhead) contains isoforms RCC1L^V1^ and RCC1L^V2^, while the 37 kDa band (red arrowhead) corresponds to isoform RCC1L^V3^. Steady-state levels of mtLSU and mtSSU proteins, including mitochondrial ribosomal proteins and biogenesis factors, were analysed. MT-CO1, MT-CO2 and GAPDG proteins were used as mitochondrial-encoded and loading control, respectively. (**B**) [^35^S]-methionine *de novo* synthesis of mitochondrially encoded proteins in mock, AllStars and RCC1L silenced HEK cells. Newly synthesized proteins were visualized after exposure of the dried gel to phosphorscreens (above) and signal intensity was quantified with ImageQuant software. Coomassie stain signal was used as loading control for normalisation (below). (**C**) Quantitative PCR (qPCR) analysis of the steady state levels of rRNAs (16S and 12S) and a representative mtDNA-encoded mRNAs (MT-CO1) normalised to GAPDH in mock, AllStars and RCC1L silenced HEK cells. In panels (B-C), data represent mean ± SD from three independent experiments. t-test: *P < 0.05, **P < 0.01, ***P < 0.001. See S4 Table for quantitative data in this figure.

The overexpression of RCC1L^V1^ and RCC1L^V3^ isoforms showed an association of each isoform with a different subunit of the mitoribosome and changes in steady state levels not only of structural subunits but also of proteins involved in their assembly. Therefore, we aimed to analyse the effect RCC1L loss in the mitoribosome biogenesis. First, we analysed the effect of RCC1L knock down on each isoform-specific mRNA (S8 Fig). Silencing of RCC1L^V1^ was very effective with RNAi 1–3 and 9–10 and only slightly less effective with RNAi 7–8. Silencing of RCC1L^V2^ was achieved with RNAi 7–10 and minor changes were also detected when non isoform specific RNAi 2–3 or 5–6 were used, most likely due to a secondary effect or non-specific targeting. Although silencing of RCC1L^V3^ was never so efficient as for the other two isoforms, mRNA levels were down to 40–50% of controls, except in the case of RNAi 6 were only a 20% reduction was achieved. Then, we analysed the effect of RCC1L knock down on protein steady state level when one or more isoforms were knocked down (Fig 6A). RNAi 1–3 exclusively targeted RCC1L^V1^ and a clear reduction in the levels of this isoforms, without a significant change in the 37 kDa RCC1L, was detected. Moreover, significant decrease in other proteins was also observed, namely structural components of the mtLSU (uL3 and bL12) and pseudouridylation module (TRUB2, RPUSD3 and RPUSD4). Interestingly, a significant increase in the levels of GTPBP10 was detected while the other two GTPases involved in mtLSU assembly, MTG1 and MTG2, remained unchanged (Fig 6A and S9 Fig). Co-silencing of RCC1L^V1^ and RCC1L^V2^ with RNAi 7–8 was very similar to silencing of RCC1L^V1^ alone albeit slightly less efficient, suggesting that RCC1L^V2^ may not have an effect on these proteins (Fig 6A and S9 Fig). Silencing of RCC1L^V3^ was attempted with RNAi 4–6 and results showed that it was more difficult to silence this isoform than RCC1L^V1^, and indeed RNAi 6 was only marginally effective (Fig 6A and S9 Fig). The decreased of RCC1L^V3^ was accompanied by a significant reduction in the levels of all the proteins related to mtSSU we analysed, while no such change was detected neither in mtLSU proteins nor in the 50 kDa RCC1L (Fig 6A and S9 Fig). When all three isoforms were silenced with RNAi 9–10, components of both the mtLSU and mtSSU were decreased, with the exception of GTPase GTPBP10 that was slightly increased and GTPases MTG1 and MTG2 that remained unaltered (Fig 6A and S9 Fig). Silencing of the different RCC1L isoforms seemd to produce changes in steady state levels of mitoribosome proteins that were in the same direction as the overexpression (Fig 2A and 2B) implying a tight regulation of the levels of RCC1L is required for proper mitoribosome assembly.

The observed reduction in steady state levels of mitoribosome proteins and mitochondrially encoded proteins MT-CO1 and MT-CO2 (Fig 6A and S9 Fig) prompted us to investigate the effect of RCC1L silencing on mitochondrial translation. *De novo* mitochondrial protein synthesis was significantly decreased by about 60% compared to controls for all RNAi (Fig 6B), except for RNAi 6 where it just decreased a 30%, as in agreement with the level of RCC1L silencing achieved for that RNAi (Fig 5A). RCC1L silencing was affecting the synthesis of all mitochondrial proteins to the same extent as no bias towards some proteins was detected (Fig 6B), supporting the idea that it was interfering in a common general process such as ribosome assembly and stability. Indeed, we have observed that silencing of RCC1L^V1^ significantly decreased the levels of both 16S rRNA and proteins associated with the mtLSU (Fig 6A and 6C) while silencing of RCC1L^V3^ slightly altered the levels of 12S rRNA and had a greater effect on the proteins associated with the mtSSU (Figs 6A and 5C), while no effect on mitochondrial DNA copy number was observed (S10 Fig). Hence, mtLSU or mtSSU were depleted in each case, and therefore, the amount of fully assembled mitoribosome that could be formed was reduced and as a consequence so was mitochondrial translation.

**Fig 7:**
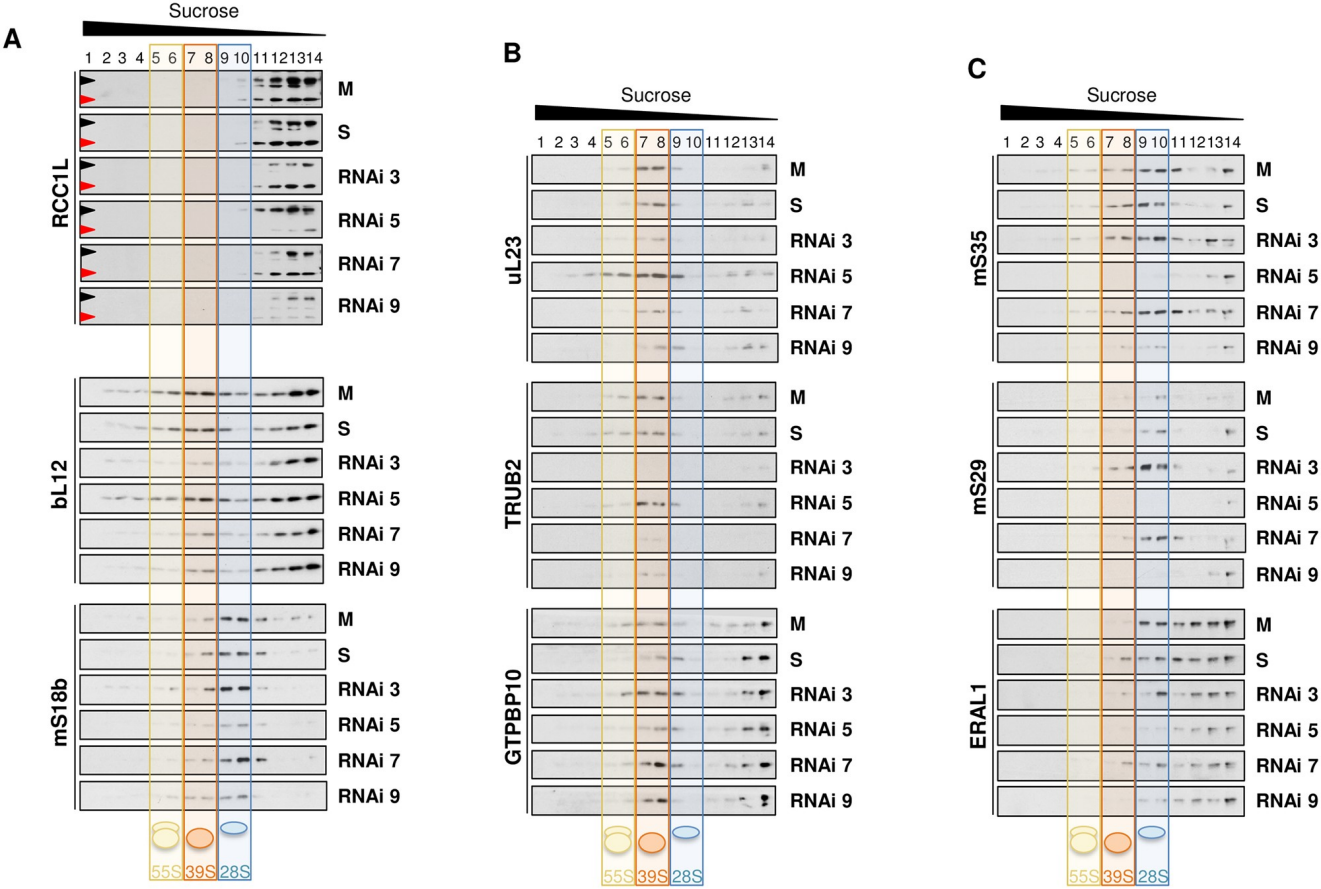
RCC1L silencing affect mitochondrial ribosome assembly. (**A**) Mitochondrial ribosome profile in experiments of silencing of RCC1L^V1^ (RNAi 3) and RCC1L^V3^ (RNAi 5) isoforms and combined silencing of RCC1L^V1^ and RCC1L^V3^ (RNAi 7) and all three isoforms (RNAi 9) in HEK cells. Mock (M) and AllStars dsRNA (S) transfected cells were used as negative controls. Equal amounts of cellular lysates from each of the four cell lines were separated on a 10–30% (v:v) isokinetic sucrose gradient and fractions were analysed by immunoblotting. In the immunoblots of endogenous RCC1L, the 50 kDa band (black arrowhead) contains isoforms RCC1L^V1^ and RCC1L^V2^, while the 37 kDa band (red arrowhead) corresponds to isoform RCC1L^V3^. Antibodies against structural components of mtLSU (bL12) and mtSSU (mS18b) were used for mapping the peak fractions of each mitochondrial ribosomal subunit. (**B**) Mitochondrial ribosome profile for mtLSU proteins (uL23) and mitochondrial ribosome biogenesis factors (TRUB2 and GTPBP10) in experiments of RCC1L silencing. (**C**) Mitochondrial ribosome profile for mtSSU proteins (mS35 and mS29) and mitochondrial ribosome biogenesis factor (ERAL1) in experiments of RCC1L silencing. Transparent blue, orange and yellow colours mark the fractions where the 28S mtSSU (fractions 9–10), 39S mtLSU (fractions 7–8) and 55S monosome (fractions 5–6) peak, respectively whereas non-assembled subunit peaks are left unmarked (fractions 11–14). Equal volume of each fraction was loaded for all cell lines.

In order to get further information on the effect of RCC1L silencing on mitoribosome subunit assembly, we performed sucrose gradient sedimentation analysis on cellular extracts on those RNAi that better knocked down RCC1L: RNAi 3, RNAi 5, RNAi 7 and RNAi 9 (Fig 6A). Silencing of a single RCC1L isoform did not significantly alter the state levels of the other isoforms, as already observed in the whole cell lysates, nor their distribution along the gradient (Fig 7A). Silencing of isoform RCC1L^V1^ (RNAi 3, 7 and 9) significantly decreased mtLSU (fractions 7 and 8) and also, albeit to a much lesser extent, the sub-mitoribosomal fractions (fractions 11–14), as shown for bL12 and uL23 (Fig 7A and 7B). The pseudouridylation module protein TRUB2 mirrored the changes of mtLSU ribosomal proteins (Fig 7B). Unexpectedly, the GTPase GTPBP10 was found to be significantly increased in mtLSU fractions (fractions 7 and 8), confirming the overall increase detected previously (Fig 6A). The accumulation of GTPBP10 in mtLSU fractions could be due to: (i) lack of substrate but also in the overexpression of RCC1L^V1^ lower levels of mtLSU were obtained and no increase but a decrease of GTPBP10 was observed; (ii) most likely GTPBP is accumulated in its inactive form due to the lack of RCC1L. Silencing of isoform RCC1L^V3^ (RNAi 5 and 9) significantly decreased mtSSU (fractions 9 and 10) and to some extend also the sub-mitoribosomal fractions (fractions 11–14), as revealed by mS18b and mS35 (Fig 7A and 7C). The structural subunit mS29, that has intrinsic GTPases activity, and the GTPase ERAL1did show the same pattern as the other two structural subunits we analysed, mS18b and mS35 (Fig 7A and 7C). Focusing on the GTPases, it is worth to stress the opposite effect overexpression and silencing had on them: GTPBP10 was decreased when RCC1L^V1^ was overexpressed, while it was increased when the same isoform was silenced, despite the fact that in both cases mtLSU was decreased. By contrast, ERAL1 was increased in RCC1L^V3^ overexpression but decreased when it was silenced, always leading to decreased mtSSU (Figs 4 and 7).

### RCC1L^V1^ binds uridine homopolymer and RCC1L^V2^ binds thymidine homopolymer

**Fig 8:**
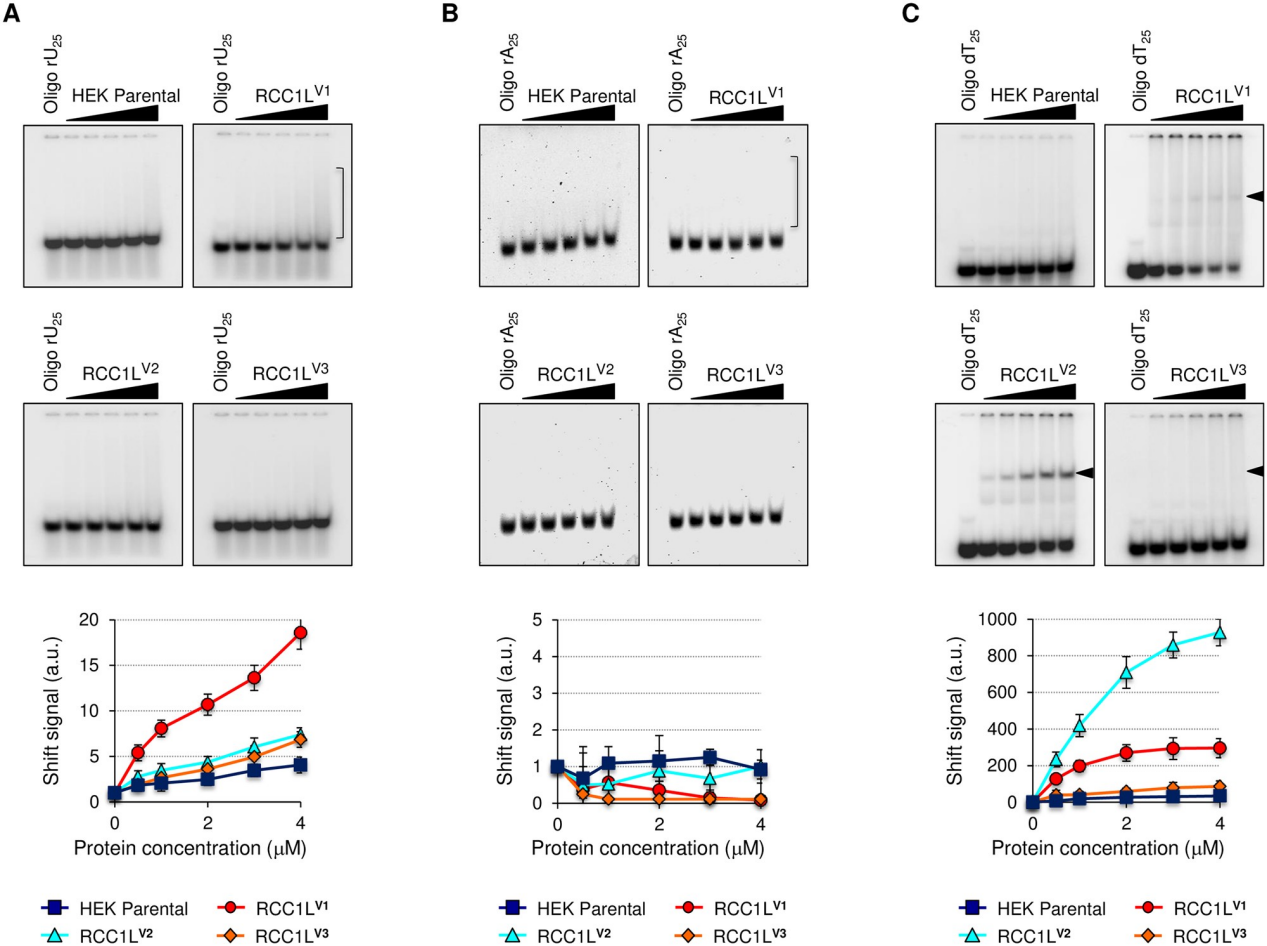
Nucleic acid binding properties of RCC1L isoforms. (**A**) Electrophoretic mobility shift assay (EMSA) using 10 nM [^32^P]-end-labelled RNA 25-mer polyuridine (rU_25_) oligo for 30 min at 37°C (above). Densitometric analysis was based on quantification of the s ignal in the region marked with a bracket and using the no-protein lane as reference (below). (**B**) Electrophoretic mobility shift assay (EMSA) using 10 nM [γ^32^P]-end-labelled RNA 25-mer polyadenosine (rA_25_) oligo for 30 min at 37°C (above). Densitometric analysis was based on quantification of the signal in the region marked with a bracket and using the no-protein lane as reference (below). (**C**) Electrophoretic mobility shift assay (EMSA) using 10 nM [γ^32^P]-end-labelled DNA 25-mer polydeoxythymidine (dT_25_) oligo for 30 min at 37°C (above). Densitometric analysis was based on quantification of the signal in the region marked with a filled arrowhead and using the no-protein lane as reference (below). In panels (A-C), increasing concentrations of dialysed recombinant RCC1L proteins (0–4 μM) were used. Equivalent volumes of HEK parental mitochondrial dialysed elution were used as negative control in each experiment. Data represent mean ± SD from two independent experiments. See S5 Table for quantitative data in this figure.

RCC1L has been previously identified as a component of the mitochondrial pseudouridylation module [19] [20] [21] [11]. Therefore, we checked if RCC1L isoforms were able to bind RNA, and in particular uridine. When we used a 25-mer uridine homopolymer in EMSA experiments, we detected a weak diffuse signal, albeit proportional to the amount of protein, in RCC1L^V1^ (Fig 8A). This would be suggestive of some kind of interaction between this isoform or its binding partners with uridine residues and in agreement with its proposed role in pseudouridylation of 16S rRNA [19] [20] [21] [11]. No significant difference compared to the negative control (HEK parental) was observed for the other two isoforms (Fig 8A). The specificity of this interaction was demonstrated by the lack of interaction between any of the three isoforms and a 25-mer adenosine homopolymer (Fig 8B). However, the most striking result was the unexpected binding of RCC1L^V2^ to a 25-mer deoxythymidine homopolymer, which resulted in a clear shifted band even at the lowest protein concentration (Fig 8C). This strong interaction with DNA may also be responsible for the distinctive membrane-like behaviour we observed above (Fig 1E) could explain the sharp differences we have observed at every single level between this isoform and the other two. RCC1L^V1^ also presented a significant but weaker shift with the 25-mer deoxythymidine homopolymer, while RCC1L^V3^ had a much weaker signal but still over two-fold higher compared to HEK parental (Fig 8C). A weaker and differential affinity of these isoforms to bind the substrate compared to RCC1L^V2^ and the presence of “contaminant” RCC1L^V2^ in different levels in the protein extract of each of the other two isoforms could explain these results. While we cannot rule out the first explanation, the presence of other isoforms in the pull downs of each single overexpressed isoform was already shown (Fig 5A, 5C and 5D), giving further support to the second hypothesis.

## Discussion

One of the most important breakthroughs of mitoribosome assembly in recent years relates to the realisation that this process involves not a single but three mitochondrial matrix sites or foci, namely the mitochondrial nucleoids, the RNA granules and the RNA degradosome [7]. These foci are dynamic overlapping structures and therefore, it is likely to find proteins involved in mitochondrial ribosome assembly also interacting with mitochondrial nucleoids. Hence, it is not surprising to find RCC1L isoforms being in the matrix and having an association with the mitochondrial inner membrane similar to nucleoid, RNA granule and ribosomal proteins like TFAM, mS18b or uL11. Furthermore, RCC1L^V1^, or its close interactors, showed some RNA binding capacity on polyuridine substrates, while RCC1L^V2^ displayed a strong affinity for DNA binding. This difference could be indicative of the uncoupling between the nucleoid and the RNA granule roles, i.e., since more than one isoform is present in human mitochondria, each of them may have specialised and retained only part of the features present in species where only one isoform is found. RCC1L^V2^ has retained the DNA binding capacity but it seems not to have a clear role in mitoribosome assembly, while RCC1L^V1^ and RCC1L^V3^ are essential for mitoribosome biogenesis. In addition, a small proportion of all three isoforms seem to interact with each other, suggesting that their roles are still interconnected and that this interaction may be essential for their function. In support of this idea, other proteins that have only one isoform show both nucleoid and mitoribosome functions. The GTPase C4ORF14, while co-purifying with TFAM and able to bind DNA, co-fractionated with mtSSU components in sucrose gradients and was also able to bind RNA [14]. Similarly, ATAD3 was described as a DNA binding protein whose silencing altered the topological state of mtDNA [23] and also as a binding partner of the mitochondrial ribosome localising in the RNA granules [24] [25].

Three different RCC1L isoforms are reported in databases and our study provides evidence that all of them are present in mitochondria but may have different roles. RCC1L^V1^ is the most abundant RCC1L isoform and can be overexpressed to high levels. Altered gene expression of this isoform, either by overexpression or silencing, resulted in decreased mtLSU biogenesis, affecting the steady state level of some MRPs and decreasing translation. While the precise role of RCC1L^V1^ in mtLSU biogenesis is not known, it has been proposed that RCC1L^V1^ is a component of the pseudouridylation module, a set of proteins involved in the modification of 16S rRNA [20]. Indeed, other components of the pseudouridylation module (TRUB2, RPUSD3, RPUSD4) are downregulated in silenced RCC1L^V1^ and, in the same way, RCC1L has been described to be downregulated in TRUB2 depleted cells [26]. As a consequence, 16S rRNA but not 12S rRNA levels are decreased, as reported in non-isoform specific RCC1L silencing experiments [20]. By contrast, RCC1L^V1^ overexpression increased the levels of the pseudouridylation module proteins we analysed, highlighting the parallel behaviour of all the proteins from this module. Despite this increase, 16S rRNA levels were still significantly lower than in the overexpression of the other isoforms, suggesting that both hypo- and hyperpseudouridylation of 16S RNA may be detrimental for its stability. Furthermore, co-immunoprecipitation experiments have shown an interaction between RCC1L^V1^ and proteins from the pseudouridylation module, in agreement with previous observations [20]. Similarly, RCC1L has also been found in the pull downs of pseudouridylation proteins such as NGRN [20], TRUB2 and FASTKD2 [26]. This involvement in 16S rRNA pseudouridylation may relate to the observed weak but significant binding of RCC1L^V1^ to polyuridine but not polyadenosine not deteceted in the other two isoforms. However, RCC1L^V1^ does not contain any RNA modifying domain and therefore its effect on 16S rRNA might be through the interaction with pseudouridylation proteins. There are two possible scenarios for this interaction: (i) RCC1L^V1^ directly regulates the steady-state levels and/or activity of pseudouridylation proteins by an unknown mechanism or (ii) most likely RCC1L^V1^, through its GDP/GTP nucleotide exchange factor activity [22], regulates certain mtLSU GTPases that would act upon pseudouridylation proteins. Out of the three GTPases described to be involved in mtLSU biogenesis, MTG1, MTG2 and GTPBP10, our results pointed towards the latter as the most likely candidate. GTPBP10 was the most enriched GTPase in RCC1L^V1^ pull downs (five- and four-fold higher than MTG1 and MTG2, respectively), despite the fact that the levels of GTPBP10 bound to mtLSU were decreased in RCC1L^V1^ overexpression. In addition, silencing of RCC1L^V1^ led to the accumulation of GTPBP10 in mtLSU fractions. As a GTPase, GTPBP10 would hydrolyse GTP to GDP during mitoribosome assembly and RCC1L^V1^ would restore the GTP and activity of the protein. Thus, silencing of RCC1L^V1^ would prevent the recycling of GTPBP10 and as a consequence, its inactive form accumulated in the mtLSU fractions, while its overexpression may activate it at all times, even in conditions in which its inactivation is required. The mechanism by which GTPBP10 would regulate pseudouridylation proteins has not been described yet but there is some evidence linking these proteins, as follows. We have shown that after GTPBP10, the proteins with higher enrichment in RCC1L^V1^ pull downs were pseudouridylation proteins, mainly TRUB2 and RPUSD4. Quantitative mass spectrometry analysis of FLAG-tagged GTPBP10 identified TRUB2, NGRN and RPUSD4 with high scores among other proteins [11]. Most significantly, RCC1L ^V1^ was also present in the list with a high score [11], giving further support to our hypothesis. In addition, the ablation of GTPBP10 has been reported to decrease NGRN levels resulting in 16S rRNA diminishment [11] while RPUSD3 has been found increased in mtLSU fractions from GTPBP10 knock out cells [12]. In *Salmonella typhimurium*, the GTPase YhbZ (homologue of *E*. *coli* ObgE) binds to the 30S ribosomal subunit in the head region of 16S rRNA and it specifically interacts with the rRNA pseudouridylate synthase, RluD. This suggests YhbZ would be involved in delivering RluD rRNA-modifying enzyme to its site of action [27]. Since GTPBP10 is the homologue of YhbZ and ObgE, we postulate that this enzyme would be involved in bringing pseudouridylation proteins to 16S rRNA in the mtLSU and that its transition from inactive to active state would be dependent on binding to GDP/GTP provided by RCC1L^V1^.

RCC1L^V3^ is the most recently identified isoform in humans and hence, no information about it has been published so far. Our results show that it has a very similar behaviour to RCC1L^V1^ but involving mtSSU instead of mtLSU and decreasing mitochondrial translation. The GTPases associated with mtSSU (ERAL1 and mS29) were the most enriched proteins in RCC1L^V3^ co-immunoprecipitations. ERAL1 was accumulated in mtSSU and sub-mtSSU fractions when RCC1L^V3^ was overexpressed, while it was significantly decreased in silenced RCC1L^V3^. These results would imply that an excess of RCC1L^V3^ would bring additional ERAL1 to the assembling mtSSU and since ERAL1 binds the 3’ terminal stem-loop region of 12S rRNA [28], a constant presence of ERAL1 may inhibit progression of the ribosome assembly leading to its degradation. Indeed, Era, the *E*. *coli* homologue of ERAL1, binds to the 16S rRNA in a pocket that precludes the association of both ribosomal subunits (30S and 50S) into the monosome [29]. In particular, bacterial Era prevents mRNA recruitment to the 30S subunit by blocking the interaction between mRNA and 16S rRNA and thus, final activation of the 30S subunit can only be achieved after the release of Era [30]. On the other hand, silencing of RCC1L^V3^ would result in lack of recruitment of ERAL1 to the 12S rRNA, which would lead to its decay and subsequently that of mtSSU [28]. The MPR mS29 has also been proposed to be involved in mitochondrial monosome assembly by mediating the contact between mtSSU and mtLSU [4] [3], in a process that may require GTP hydrolysis. Moreover, ERAL1 has been shown to interact with mS29 and it has been speculated that this interaction takes place when they are free from the mitoribosome and relate to apoptosis and other non-ribosomal functions [13]. C4ORF14 (hNOA1) and mS29 have also been reported to interact with each other and regulate γ-interferon induced apoptosis [31]. While a role for RCC1L^V3^ in mtSSU assembly is supported by our data, the possible involvement of this isoform in apoptotic processes along with the GTPases ERAL1, mS29 and C4ORF14 will need to be further investigated.

Collectively, our results show that the RCC1L gene produces three mitochondrial isoforms and that two of them, RCC1L^V1^ and RCC1L^V3^, are essential for correct biogenesis of mitochondrial ribosome subunits. RCC1L isoforms interact with different GTPases to promote mitoribosome subunit assembly but also their expression has to be coordinated in order to balance the biogenesis of each subunit and subsequently the formation of the monosome. The presence of a third isoform, RCC1L^V2^, showing DNA binding properties but little effect on mitoribosome biogenesis is very intriguing and its characterization may contribute to a better understanding of the relationship between mitochondrial nucleoids, the RNA granules and the translation machinery.

## Materials and methods

### Cell culture and transfections

Human embryonic kidney cells HEK293T (HEK parental) and epitheloid cervix carcinoma HeLa cell lines were maintained in high-glucose medium (Gibco) supplemented with 10% tetracycline-free FBS (Merck Biochrom), 1% penicillin-streptomycin and at 37°C in a humidified atmosphere of 5% CO_2_.

Human cDNAs corresponding to each of the three isoforms of RCC1L (WBSCR16), RCC1L^V1^, RCC1L^V2^ and RCC1L^V3^, were cloned into pcDNA5/FRT/TO plasmid (Invitrogen) with a carboxy-terminal STREP2 followed by a FLAG tag or just HA tag. Inducible transgenic HEK and HeLa cell lines were established by transfection with the STREP2-FLAG constructs using Lipofectamine 2000 (Invitrogen). Control and transgenic cell lines were cultured in the presence of 15 μg/ml of blasticidin and 100 μg/ml of Zeocin or 100 μg/ ml of hygromycin and 15 mg/ml of blasticidin, respectively. Transgene expression was induced with 10 ng/ml doxycycline for 3–6 days and cells were collected or mitochondria purified for further analysis [32]. When required, a second transfection using Lipofectamine 2000 was carried out for transient expression of HA-tagged RCC1L isoforms in induced STREP2-FLAG cell lines. In this case, cells were collected for analysis 3 days after transfection.

For silencing experiments, HEK parental cells were transfected with INTERFERin (Polyplus) and 50 nM Stealth^™^ RNAi duplexes (Invitrogen). Ten different Stealth RNAi were used (S1 Table): three isoform RCC1L^V1^ specific (RNAi 1–3), three isoform RCC1L^V3^ specific (RNAi 4–6), two targeted both isoform RCC1L^V1^ and RCC1L^V2^ (no specific target for isoform RCC1L^V2^ could be found) (RNAi 7–8) and two hit all three isoforms (RNAi 9–10). Mock transfections and AllStars dsRNA negative control (Qiagen) were used as controls. After 3 days, cells were either lysed and RNA, DNA and total cellular proteins extracted or they were used for analysis of mitochondrial translation.

### Cell fractionation

Cellular fractionation experiments in HEK parental and HeLa cells was performed with ProteoJET Cytoplasmic and Nuclear Protein Extraction Kit (Fermentas, K0311) according to the manufacturer’s instructions. In our case, mitochondria were recovered from the cytoplasmic fraction by centrifugation at 8000g_max_ for 10 min. Cell lysates along with cytosolic, mitochondrial and nuclear fractions were loaded onto 4–12% polyacrylamide gels and analysed by immunoblotting. GAPDH, COX2 and LBR were used as markers for the cytosolic, mitochondrial and nuclear fractions.

### Confocal microscopy

Parental and transfected HeLa cells were grown on collagen-coated glass coverslips and induction of transgene expression was carried out with 10 ng/ml doxycycline for 24 h. Cells were washed and live-stained with 100 nM MitoTracker Red. After fixation and permeabilization, cells were incubated with mouse monoclonal anti-FLAG antibody M2 (1:1000 Sigma) followed by Alexa-Fluor 488 goat anti-mouse secondary antibody (1:1000, Invitrogen). Coverslips were mounted with ProLong Gold antifade reagent containing 4’,6-diamidino-2-phenylindole dihydrochloride (DAPI; Invitrogen). Images were acquired with a Nikon 63x oil immersion objective in a A1R-A1 Nikon N-SIM confocal microscope.

### Trypsin digestion

Mitochondria from HEK parental cells (2mg/ml) purified with sucrose gradients were resuspended in either isotonic (20 mM HEPES pH 7.8, 2 mM EDTA, 210 mM mannitol, 70 mM sucrose) or hypotonic (10 mM HEPES pH 7.8) buffer left untreated or treated with 100 μg/ml of trypsin at room temperature for 30 min. Purified mitochondria were alternatively treated with 200 or 500 mg/ml digitonin in isotonic buffer for 10 min at 4°C prior to trypsin treatment [32]. After washing and pelleting mitochondria three times, the organelles were lysed with 1% SDS in isotonic buffer. Samples were loaded onto 4–12% polyacrylamide gels and analysed by immunoblotting. TOM70, CYC, SDHB and CS were used as markers for the mitochondrial outer membrane, intermembrane space, mitochondrial inner membrane and mitochondrial matrix, respectively.

### Extraction of mitochondrial membranes

Sucrose-gradient purified mitochondria from HEK parental cells (2 mg/ml) were sonicated for 1 min on ice followed by centrifugation at 10 000g_max_ for 10 min at 4°C. The supernatant was treated with either 150 or 500 mM NaCl or 2% SDS on ice for 30 min followed by centrifugation at 122 000g_max_ for 30 min at 4°C. Supernatants containing the soluble fractions were recovered and pellets with membrane-associated proteins were suspended in equal volume of isotonic buffer containing 0,2% SDS [33]. Samples were loaded onto 4–12% polyacrylamide gels and analysed by immunoblotting. TOM70 and CS were used as controls of integral membrane and soluble protein, respectively.

### Iodixanol and isokinetic sucrose gradients

Mitochondria was purified from HEK parental cells and cells induced with 10 ng/ml doxycycline for 3 days to express different isoforms of RCC1L. For iodixanol gradients, purified mitochondria (2 mg) were lysed and loaded onto 7.2 ml 20–45% (v/v) self-forming continuous iodixanol gradients [32]. After centrifugation at 100 000g_max_ for 14 h at 4°C, 18 fractions of 450 μl were collected and analysed by Western blotting. Alternatively, samples (400 μg) were loaded onto 13 ml 10–30% (v/v) linear sucrose gradients and centrifuged for 16 h at 100 000g_max_ at 4°C [14]. Fractions of 400 μl were collected and analysed by Western blotting. In the case of RCC1L silencing, total cell lysates (0.5 mg) were loaded onto 2 ml 10–30% (v/v) linear sucrose gradients and 20 factions of 100 μl were collected analysed by immunoblotting [34]. In both cases, fractions from the different gradients were run, immunoblotted and exposed in a way that would allow direct comparison of the signal in each gradient. Two biological replicates were performed for each gradient.

### Immunoblotting

Primary antibodies used in this study were: mouse anti-GAPDH (Abcam, ab8245), rabbit anti-MT-CO2 (Abcam, ab79393), rabbit anti-RCC1L (Proteintech, 13796-1-AP), rabbit anti-LBR (Proteintech, 12398-1-AP), mouse anti-TOMM70 (Abcam, ab89624), mouse anti-CYC (Proteintech, 66264-1-Ig), mouse anti-SDHB (Abcam, ab14714), rabbit anti-CS (Proteintech, 16131-1-AP), rabbit anti-TFAM (Abcam, ab131607), rabbit anti-mS18b (Proteintech, 16139-1-AP), rabbit anti-uL11 (Sigma, SAB2701374), rabbit anti-GARS (Abcam, ab42905), mouse anti-FLAG (Sigma, F1804), mouse anti-ACO2 (Abcam, ab110321), rabbit anti-bL12 (Proteintech, 14795-1-AP), rabbit anti-ICT1 (Proteintech, 10403-1-AP), rabbit anti-MALSU1 (Sigma, HPA020487), rabbit anti-MTG1 (Novus, NBP2-19428), rabbit anti-MTG2 (Proteintech, 20133-1-AP), rabbit anti-GTPBP10 (Novus, NBP1-85055), mouse anti-mS29 (Abcam, ab11928), rabbit anti ERAL1 (Proteintech, 11478-1-AP), rabbit anti-C4orf14 (abcam, ab101348), rabbit anti-VDAC1(Abcam, ab15895), mouse anti-ATPA5 (Abcam, ab14748), mouse anti-UQCRC2 (Abcam, ab14745), mouse anti-MT-CO1 (Abcam, ab14705), rabbit anti-uL3 (Sigma, HPA043665), rabbit anti-uS17 (Proteintech, 18881-1-AP), mouse anti-HA (Abcam, ab18181), rabbit anti-MDDX28 (Abcam, ab70821). Secondary antibodies were anti-rabbit IgG HRP (Promega, W4011), anti-mouse IgG HRP (Promega, W4021) or anti-goat IgG HRP (Promega, V8051).

### DNA and RNA analysis

Total DNA and RNA were extracted from whole cells using Wizard Genomic DNA Purification Kit (Promega) and Trizol (Invitrogen), respectively. RNA was then treated with DNase I (DNA-free kit, Ambion) and reverse transcribed with Omniscript reverse transcription kit (Qiagen). Quantitative polymerase chain reaction (qPCR) analyses were performed with TaqMan assays (Applied Biosystems): MT-CO1 (Hs02596864_g1), MT-CYB (Hs02596867_s1), MT-ND1 (Hs02596873_s1), MT-RNR1 (12S rRNA, Hs02596859_g1), MT-RNR2 (16S rRNA Hs02596860_s1), APP (Hs02339796_cn) and GAPDH (Hs02758991_g1). Relative mtDNA copy number was calculated as average of MT-CO1, MT-CYB and 12S rRNA normalised to APP. For RNA transcript levels, each gene was normalised to GAPDH. In silencing experiments, isoform-specific transcript levels were analysed by SYBR Green qPCR with the following primers: CACTGGCACTCCTTGGCAGA and GGGACCTTGCCCTGATCCTC for RCC1L^V1^, CCCGAAGAGCTGTGGAGACC and ACCCTGCTCCTCGGTCACAG for RCC1L^V2^, GGCCACAGAGCTGAGTCATCC and TTGGCTGGAGAAGGCAGAGC for RCC1L^V3^ and GGATTTGGTCGTATTGGG and GGAAGATGGTGATGGGATT for GAPDH. Each transcript was normalised to GAPDH.

### Mitochondrial translation

HEK parental cells, STREP2-FLAG tagged RCC1L expressing cells induced with 10 ng/ml doxycycline for 3 or 6 days and RCC1L silenced HEK cells after 3 days of transfection with dsRNA, along with their controls, were subjected to metabolic labelling of mtDNA encoded proteins. [^35^S]-methionine was added to the medium after treatment with emetine dihydrochloride and labelling was performed for 1 h, as previously described [35]. Cells were lysed and proteins (30 μg) were loaded onto 10–20% polyacrylamide gels. Gels were stained with Coomassie blue, dried and then exposed to Typhoon phosphorscreens, with products visualized and quantified with ImageQuant software (GE Healthcare).

### Immunoprecipitation

Sucrose gradient-purified mitochondria were obtained from HEK parental and STREP2-FLAG tagged RCC1L expressing cells induced with 3–10 ng/ml doxycycline for 3–4 days. After trypsin treatment (50 μg/ml for 30 min), mitochondria were lysed with 0.8% n-dodecyl-β-d-maltoside (DDM) and incubated overnight with 200 μl of Srep-Tactin Sepharose beads (iba). A total of 10 washes were performed before elution with 10 mM desthiobiotin in washing buffer, as previously described [14]. Input, washes and elution were loaded onto 4–12% polyacrylamide gels and analysed by immunoblotting. When required, Sepharose beads were treated with DNase I (20U/ml, Ambion), RNase A (40 μg/ml, Qiagen) or Benzonase (12.5 U/μl, Novagen) for 1 h at 4°C and washed 10 times before elution. DNA and RNA were isolated from the elution and their relative amount normalised to the untreated sample quantified by qPCR with TaqMan assays, MT-CO1 for DNA and MT-RNR1 for RNA.

### EMSA

Two homopolymer RNA oligonucleotides, rU_25_ and rA_25_ and one DNA oligonucleotide, dT_25_, were end labelled with [γ^32^P]-ATP using T4 polynucleotide kinase (New England Biolabs) and purified with G-50 columns (GE Healthcare). RCC1L^V1-3^ isoforms were partly purified from sucrose gradient-purified mitochondria using Srep-Tactin Sepharose beads (iba), as described above. Elution fractions were dialysed overnight at 4°C in 20 mM Tris-HCl pH 8.0, 10 mM MgCl_2_, 1 mM EDTA, 1 mM EGTA, 2 mM DTT, 1 mM PMSF, 50% glycerol. HEK parental sucrose gradient-purified mitochondria followed the same procedure and it was used as negative control. Increasing concentrations of dialysed protein (0.5, 1, 2, 3, 4 μM) were incubated with 10 nM [γ^32^P]-end-labelled oligonucleotide for 30 min at 37°C in binding buffer (10 mM Tris.HCl pH 9.4, 75 mM glycine, 0.1 m M EDTA, 7.5 mM MgAc2, 0.5 mg/ml BSA, 2 mM DTT, 10% glycerol). The mixtures were loaded onto native 6% polyacrylamide gels with 10 mM Tris-HCl pH 9.4, 75 mM glycine, 0.1 mM EDTA and run at 80V for 1.5 h. Gels were then dried and exposed to Typhoon phosphorscreens, with products visualized and quantified with ImageQuant software (GE Healthcare).

### Statistical analysis

All experiments were done in duplicate or at least triplicate, when quantifications were required. X-ray films were digitalised and analysed with ImageJ software and data analyses were performed in Microsoft Excel. The data are presented as mean ± standard deviation (SD) or percentages of control. Student’s two-tailed unpaired t-test were used for comparisons.

## Supporting information

S1 Fig Presence of RCC1L isoforms in mammalian species. The presence of RCC1L isoforms was carried out in in Ensemble (http://www.ensembl.org/index.html). Representative mammalian species (common names on the left), representing nine different orders (on the right) and one outgroup species (chicken) were taken into consideration. Distinction between prosimian primates and anthropoid primates is marked in the figure. Red and white boxes indicate the presence or absence, respectively, of the isoform in each species based on Ensemble data. Pink boxes mean that the isoform is not reported in Ensemble for that species but it is in other species that share a common ancestor with the former ones. Therefore, they may have not been described yet in the database or less likely, they have been lost during evolution in those species.
(TIF)

S2 Fig Mitochondrial targeting and cleavage predictions. Probability scores of mitochondrial localisation based on *in silico* predictions with different programs: Mitoprot (https://ihg.gsf.de/ihg/mitoprot.html), TargetP (http://www.cbs.dtu.dk/services/TargetP/), MitoFates (http://mitf.cbrc.jp/MitoFates/cgi-bin/top.cgi), TPred (https://tppred2.biocomp.unibo.it/tppred2) and Predotar (https://urgi.versailles.inra.fr/predotar/). The position of the predicted amino acid position cleavage for the mitochondrial targeting sequence (MTS) is also reported for each program.
(TIF)

S3 Fig Mitochondrial localisation of RCC1LV1 isoform lacking the mitochondrial targeting signal (MTS). Intra-cellular localisation of RCC1L isoforms by immunofluorescence. Parental and transfected HeLa cells expressing a STREP2-FLAG-tagged version of RCC1L^V1^ and RCC1L^V1^ lacking 37 amino acids of the predicted N-terminal mitochondrial targeting signal (MTS) were stained with DAPI for the nucleus, MitoTracker Red for mitochondria and anti-FLAG antibody followed by Alexa 488 conjugated secondary antibody for the overexpressed RCC1L proteins. Co-localisation of MitoTracker and RCC1L-specific green signal appears yellow to orange, depending on the abundance, in the merged images. Panels from HeLa and RCC1L^V1^ are the same as those shown in Fig 1.
(TIF)

S4 Fig Distribution of endogenous and overexpressed RCC1L isoforms in isokinetic sucrose gradients. Mitochondrial endogenous and overexpressed RCC1L profiles from isokinetic sucrose gradients from cells induced with 10ng/ml doxycycline for 3 days presented in Fig 4 were obtained. Traces reflect the relative abundance of the proteins in each fraction and were normalised to the levels of the same protein found in parental cell line fraction 1. In all cases, the levels of the 50 kDa RCC1L, containing RCC1L^V1^ and RCC1L^V2^ isoforms and the levels of the 37 kDa RCC1L containing RCC1L^V3^ are presented along with the FLAG signal from the overexpressed protein (absent in the case of the parental cell line). In the case of RCC1L^V3^ overexpression, RCC1L antibody allowed the quantification of endogenous and overexpressed isoform, RCC1L^V3^ and RCC1L^V3^-FLAG, respectively, on the same blot. Transparent blue, orange and yellow colours mark the fractions where the 28S mtSSU (fractions 8–9), 39S mtLSU (fractions 6–7) and 55S monosome (fractions 4–5) peak, respectively whereas non-assembled subunit peaks are left unmarked (fractions 10–14). See S6 Table for quantitative data in this figure.
(TIF)

S5 Fig RCC1L isoforms and mitochondrial ribosomal subunits in isokinetic sucrose gradients. Mitochondrial ribosome profile in parental and RCC1L overexpressing cells after induction with 10ng/ml doxycycline for 3 days. Equal amounts of mitochondrial lysates from each of the four cell lines were separated on a 10–30% (v:v) isokinetic sucrose gradient and fractions were analysed by immunoblotting. In the immunoblots of endogenous RCC1L, the 50 kDa band (black arrowhead) contains isoforms RCC1L^V1^ and RCC1L^V2^, while the 37 kDa band (red arrowhead) corresponds to isoform RCC1L^V3^. The STREP2-FLAG-tagged RCC1L proteins (RCC1L^V1^, RCC1L^V2^ and RCC1L^V3^) are also marked (grey arrowheads) in each case. Antibodies against structural components (uL3, bL19, bL27, mL40) and assembly factors (DDX28) of the mtLSU were used for immunodetection of proteins of interest. In the case of mtSSU analysis, immunoblot analysis was performed using antibodies against structural components (bS6 and mS35). Transparent blue, orange and yellow colours mark the fractions where the 28S mtSSU (fractions 8–9), 39S mtLSU (fractions 6–7) and 55S monosome (fractions 4–5) peak, respectively whereas non-assembled subunit peaks are left unmarked (fractions 10–14). Equal volume of each fraction was loaded for all cell lines.
(TIF)

S6 Fig Quantification of proteins shown in isokinetic sucrose gradients. Quantification of overall protein level of all the markers used in the isokinetic sucrose gradients presented in Fig 4 and and S5 Fig. Values are based on densitometric analysis of the entire immunoblot, containing all fractions, and normalised to loading control VDAC1 for each gradient. Data represent mean ± SD from two independent experiments. t-test: *P < 0.05, **P < 0.01. See S7 Table for quantitative data in this figure.
(TIF)

S7 Fig Pull down of STREP2-FLAG-tagged RCC1L isoforms. (**A**) Co-immunoprecipitation of STREP2-FLAG-tagged RCC1L isoforms from purified mitochondria after induction of HEK cells with 3–10 ng/ml doxycycline for 3–4 days. In the immunoblots of endogenous RCC1L, the endogenous isoforms (37 or 50 kDa band) are marked (empty arrowhead) in the elution fractions. Immunoblots for mitochondrial ribosomal proteins and biogenesis factors are presented. ACO2 was used as mitochondrial negative control for unspecific binding. Additional immunoblots not shown in Fig 4A are marked in red. (**B**) Longer exposure of the panel shown in (A). (**C**) Quantification of mitochondrial DNA and RNA by quantitative PCR (qPCR) in the elution fractions after DNAse I, RNAse A and Benzonase treatment. Data represent the average of MT-CO1, MT-CYB and 12S rRNA from two independent experiments and relative to the untreated samples. See S8 Table for quantitative data in this figure.
(TIF)

S8 Fig RCC1L isoform-specific silencing verification by qPCR. Quantification of isoform-specific mRNA levels by quantitative PCR (qPCR) in silencing experiments shown in Fig 6A and normalised to GAPDH. Silencing of RCC1L^V1^ (V1, RNAi 1–3) and RCC1L^V3^ (V3, RNAi 4–6) isoforms and combined silencing of RCC1L^V1^ and RCC1L^V3^ (V1+2, RNAi 7–8) and all three isoforms (V1+2+3, RNAi, 9–10) were performed in HEK cells. Mock (M) and AllStars dsRNA (S) transfected HEK cells were used as negative controls. Data represent mean ± SD from four independent experiments. t-test: *P < 0.05, **P < 0.01, ***P < 0.001. See S9 Table for quantitative data in this figure.
(TIF)

S9 Fig Quantification of RCC1L isoform-specific silencing effect on proteins. Quantification of protein levels in silencing experiments shown in Fig 6A and normalised to GAPDH. Silencing of RCC1L^V1^ (V1, RNAi 1–3) and RCC1L^V3^ (V3, RNAi 4–6) isoforms and combined silencing of RCC1L^V1^ and RCC1L^V3^ (V1+2, RNAi 7–8) and all three isoforms (V1+2+3, RNAi, 9–10) were performed in HEK cells. Mock (M) and AllStars dsRNA (S) transfected HEK cells were used as negative controls. Data represent mean ± SD from two independent experiments. t-test: *P < 0.05, **P < 0.01, ***P < 0.001. See S10 Table for quantitative data in this figure.
(TIF)

S10 Fig Quantification of mitochondrial DNA copy number in RCC1L silencing experiments. Quantitative PCR (qPCR) analysis of mitochondrial DNA copy number in silenced HEK cells. Silencing of RCC1L^V1^ (RNAi 1–3) and RCC1L^V3^ (RNAi 4–6) isoforms and combined silencing of RCC1L^V1^ and RCC1L^V3^ (RNAi 7–8) and all three isoforms (V1+2+3, RNAi, 9–10) in HEK cells. Mock (M) and AllStars dsRNA (S) transfected cells were used as negative controls. Data represent the average of MT-CO1, MT-CYB and 12S rRNA normalised to APP from three independent experiments. See S11 Table for quantitative data in this figure.
(TIF)

S1 Table Stealth dsRNAi list. Sequence of the Stealth dsRNAi used for RCC1L silencing.
(XLSX)

S2 Table Quantitative data of Fig 2. (XLSX)

S3 Table Quantitative data of Fig 5. (XLSX)

S4 Table Quantitative data of Fig 6. (XLSX)

S5 Table Quantitative data of Fig 8. (XLSX)

S6 Table Quantitative data of S4 Fig. (XLSX)

S7 Table Quantitative data of S6 Fig. (XLSX)

S8 Table Quantitative data of S7 Fig. (XLSX)

S9 Table Quantitative data of S8 Fig. (XLSX)

S10 Table Quantitative data of S9 Fig. (XLSX)

S11 Table Quantitative data of S10 Fig. (XLSX)
