# Supplementary figures and images for: RCC1L (WBSCR16) isoforms coordinate mitochondrial ribosome assembly through their interaction with GTPases
Source: PLoS Genet. 2020 Jul 31;16(7):e1008923. doi: 10.1371/journal.pgen.1008923 (PMC7423155; doi:10.1371/journal.pgen.1008923)

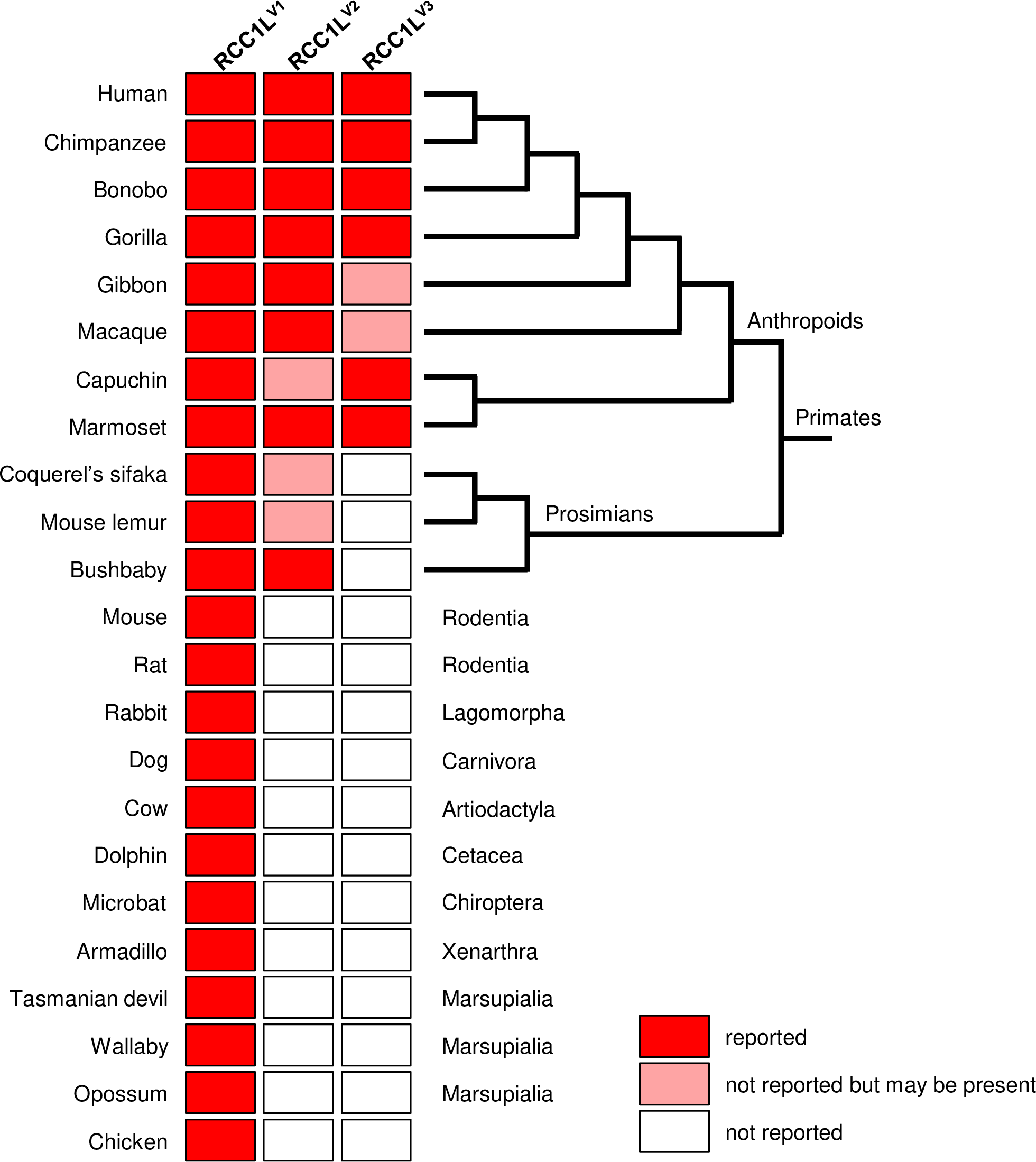

Supplement: S1 Fig — The presence of RCC1L isoforms was carried out in in Ensemble (http://www.ensembl.org/index.html). Representative mammalian species (common names on the left), representing nine different orders (on the right) and one outgroup species (chicken) were taken into consideration. Distinction between prosimian primates and anthropoid primates is marked in the figure. Red and white boxes indicate the presence or absence, respectively, of the isoform in each species based on Ensemble data. Pink boxes mean that the isoform is not reported in Ensemble for that species but it is in other species that share a common ancestor with the former ones. Therefore, they may have not been described yet in the database or less likely, they have been lost during evolution in those species. (TIF) [file pgen.1008923.s001.tif]

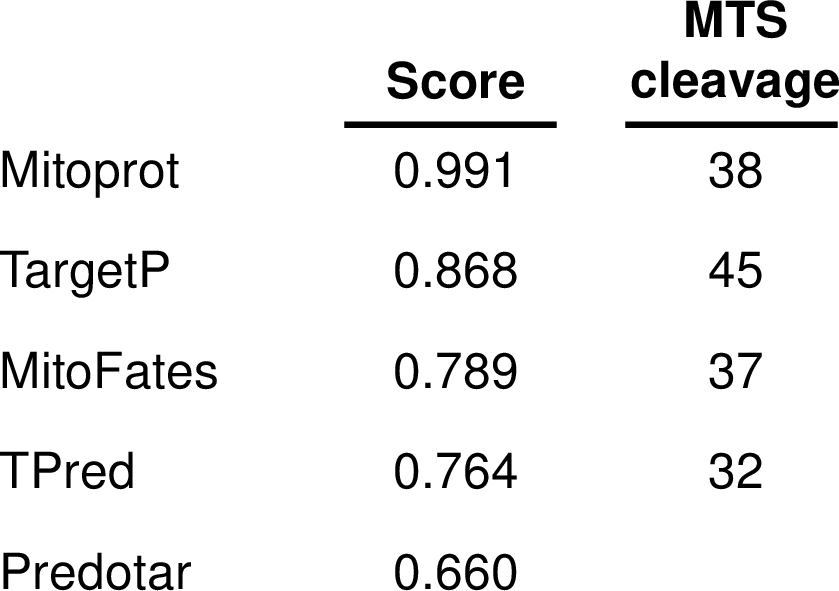

Supplement: S2 Fig — Probability scores of mitochondrial localisation based on in silico predictions with different programs: Mitoprot (https://ihg.gsf.de/ihg/mitoprot.html), TargetP (http://www.cbs.dtu.dk/services/TargetP/), MitoFates (http://mitf.cbrc.jp/MitoFates/cgi-bin/top.cgi), TPred (https://tppred2.biocomp.unibo.it/tppred2) and Predotar (https://urgi.versailles.inra.fr/predotar/). The position of the predicted amino acid position cleavage for the mitochondrial targeting sequence (MTS) is also reported for each program. (TIF) [file pgen.1008923.s002.tif]

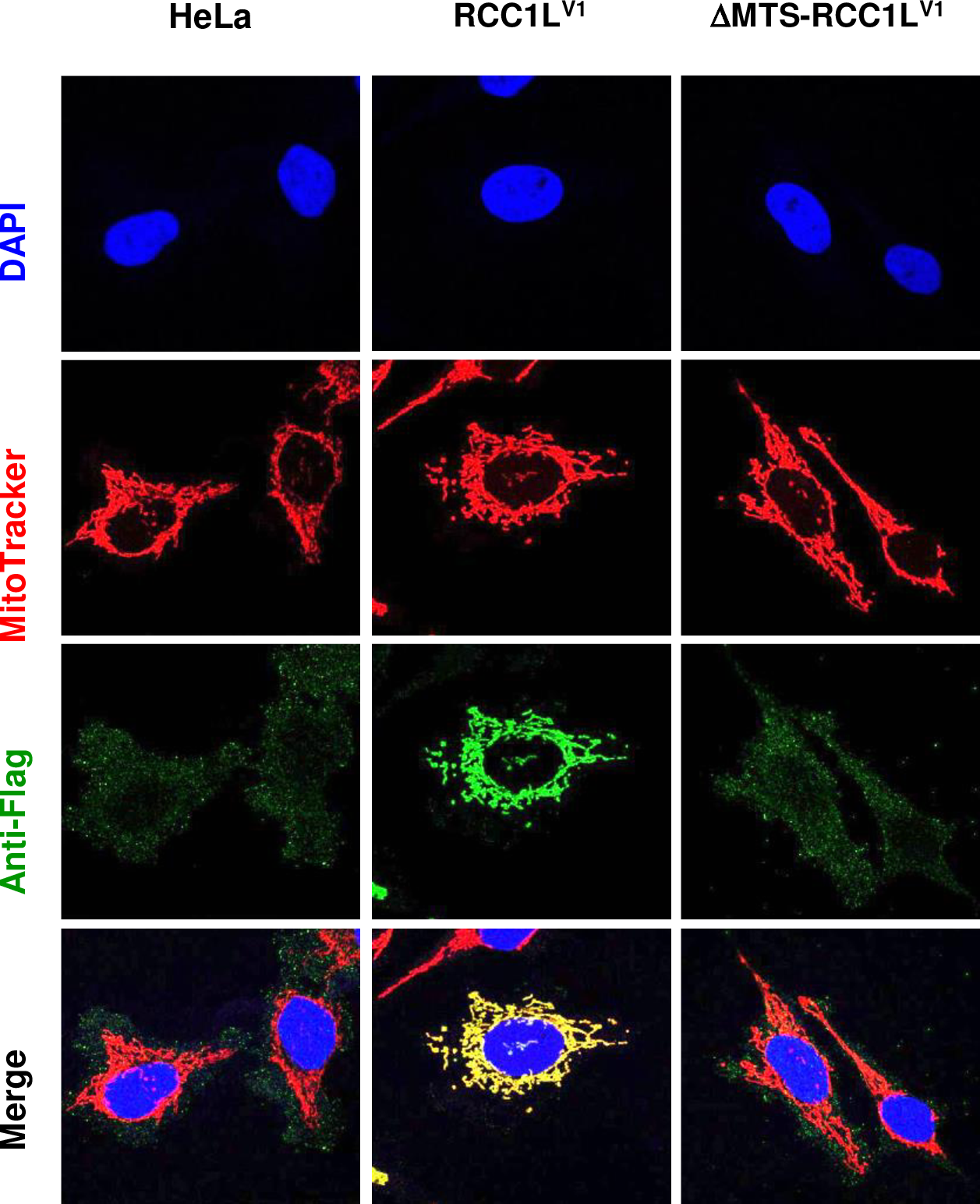

Supplement: S3 Fig — Intra-cellular localisation of RCC1L isoforms by immunofluorescence. Parental and transfected HeLa cells expressing a STREP2-FLAG-tagged version of RCC1LV1 and RCC1LV1 lacking 37 amino acids of the predicted N-terminal mitochondrial targeting signal (MTS) were stained with DAPI for the nucleus, MitoTracker Red for mitochondria and anti-FLAG antibody followed by Alexa 488 conjugated secondary antibody for the overexpressed RCC1L proteins. Co-localisation of MitoTracker and RCC1L-specific green signal appears yellow to orange, depending on the abundance, in the merged images. Panels from HeLa and RCC1LV1 are the same as those shown in Fig 1. (TIF) [file pgen.1008923.s003.tif]

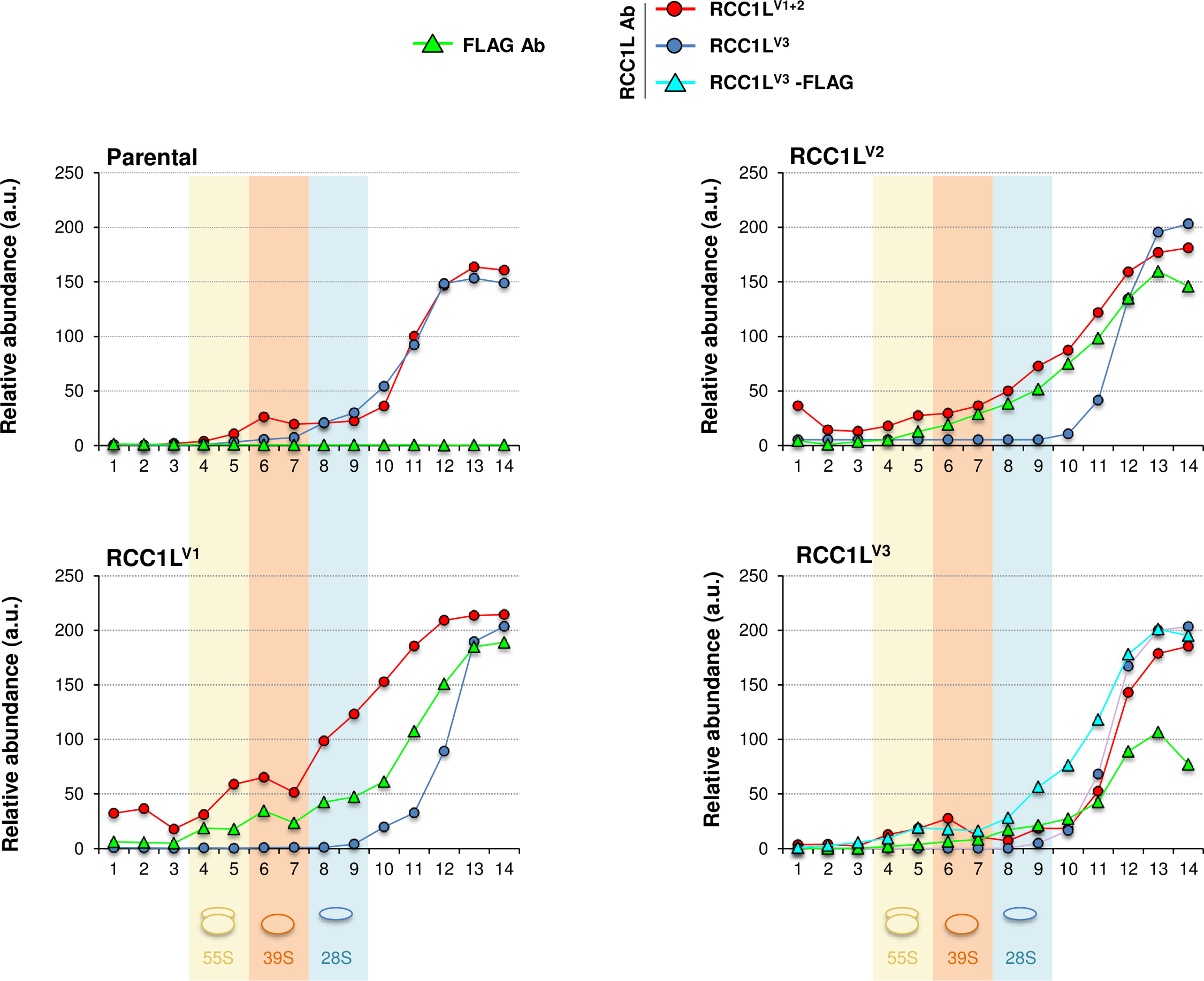

Supplement: S4 Fig — Mitochondrial endogenous and overexpressed RCC1L profiles from isokinetic sucrose gradients from cells induced with 10ng/ml doxycycline for 3 days presented in Fig 4 were obtained. Traces reflect the relative abundance of the proteins in each fraction and were normalised to the levels of the same protein found in parental cell line fraction 1. In all cases, the levels of the 50 kDa RCC1L, containing RCC1LV1 and RCC1LV2 isoforms and the levels of the 37 kDa RCC1L containing RCC1LV3 are presented along with the FLAG signal from the overexpressed protein (absent in the case of the parental cell line). In the case of RCC1LV3 overexpression, RCC1L antibody allowed the quantification of endogenous and overexpressed isoform, RCC1LV3 and RCC1LV3-FLAG, respectively, on the same blot. Transparent blue, orange and yellow colours mark the fractions where the 28S mtSSU (fractions 8–9), 39S mtLSU (fractions 6–7) and 55S monosome (fractions 4–5) peak, respectively whereas non-assembled subunit peaks are left unmarked (fractions 10–14). See S6 Table for quantitative data in this figure. (TIF) [file pgen.1008923.s004.tif]

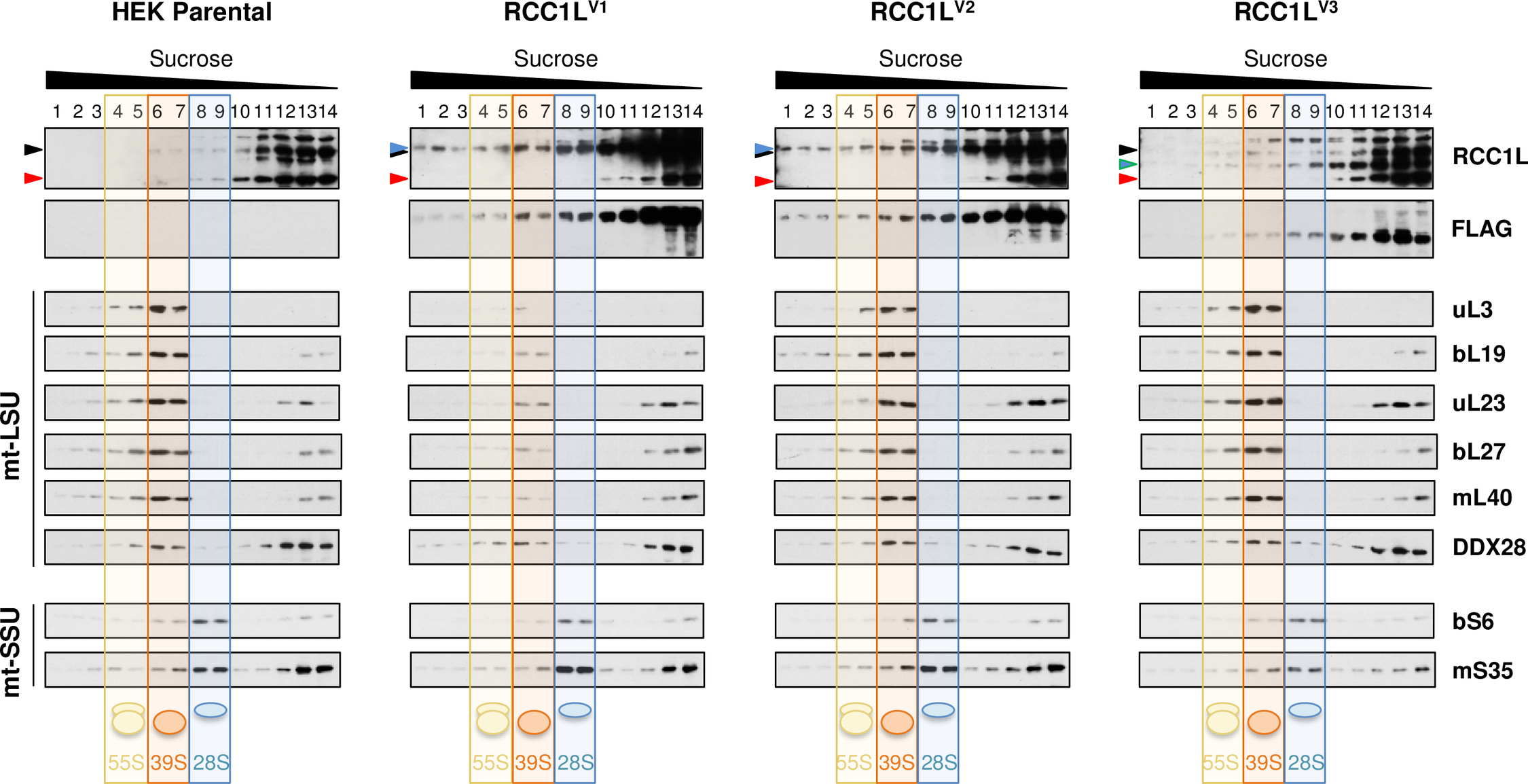

Supplement: S5 Fig — Mitochondrial ribosome profile in parental and RCC1L overexpressing cells after induction with 10ng/ml doxycycline for 3 days. Equal amounts of mitochondrial lysates from each of the four cell lines were separated on a 10–30% (v:v) isokinetic sucrose gradient and fractions were analysed by immunoblotting. In the immunoblots of endogenous RCC1L, the 50 kDa band (black arrowhead) contains isoforms RCC1LV1 and RCC1LV2, while the 37 kDa band (red arrowhead) corresponds to isoform RCC1LV3. The STREP2-FLAG-tagged RCC1L proteins (RCC1LV1, RCC1LV2 and RCC1LV3) are also marked (grey arrowheads) in each case. Antibodies against structural components (uL3, bL19, bL27, mL40) and assembly factors (DDX28) of the mtLSU were used for immunodetection of proteins of interest. In the case of mtSSU analysis, immunoblot analysis was performed using antibodies against structural components (bS6 and mS35). Transparent blue, orange and yellow colours mark the fractions where the 28S mtSSU (fractions 8–9), 39S mtLSU (fractions 6–7) and 55S monosome (fractions 4–5) peak, respectively whereas non-assembled subunit peaks are left unmarked (fractions 10–14). Equal volume of each fraction was loaded for all cell lines. (TIF) [file pgen.1008923.s005.tif]

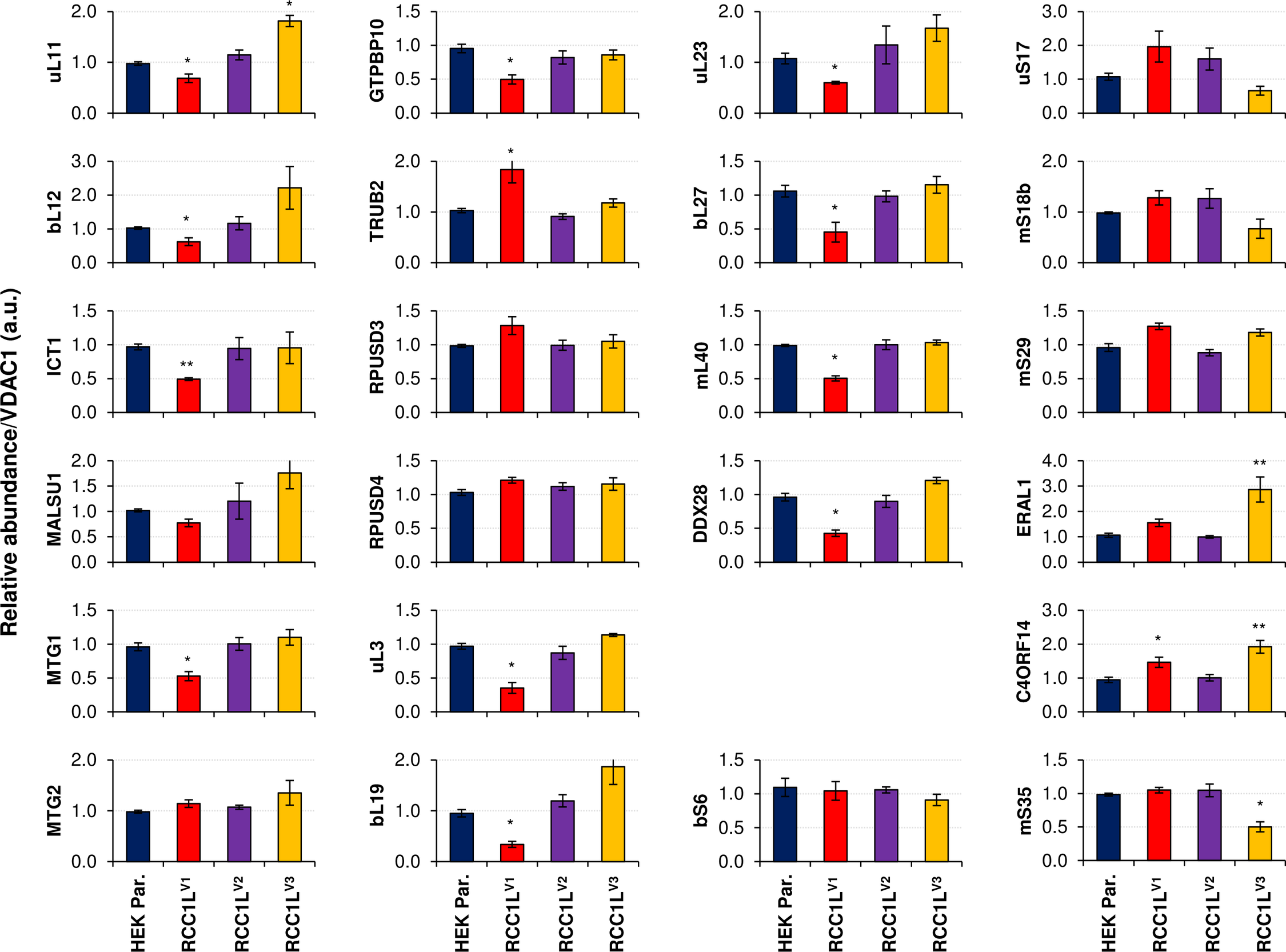

Supplement: S6 Fig — Quantification of overall protein level of all the markers used in the isokinetic sucrose gradients presented in Fig 4 and and S5 Fig. Values are based on densitometric analysis of the entire immunoblot, containing all fractions, and normalised to loading control VDAC1 for each gradient. Data represent mean ± SD from two independent experiments. t-test: *P < 0.05, **P < 0.01. See S7 Table for quantitative data in this figure. (TIF) [file pgen.1008923.s006.tif]

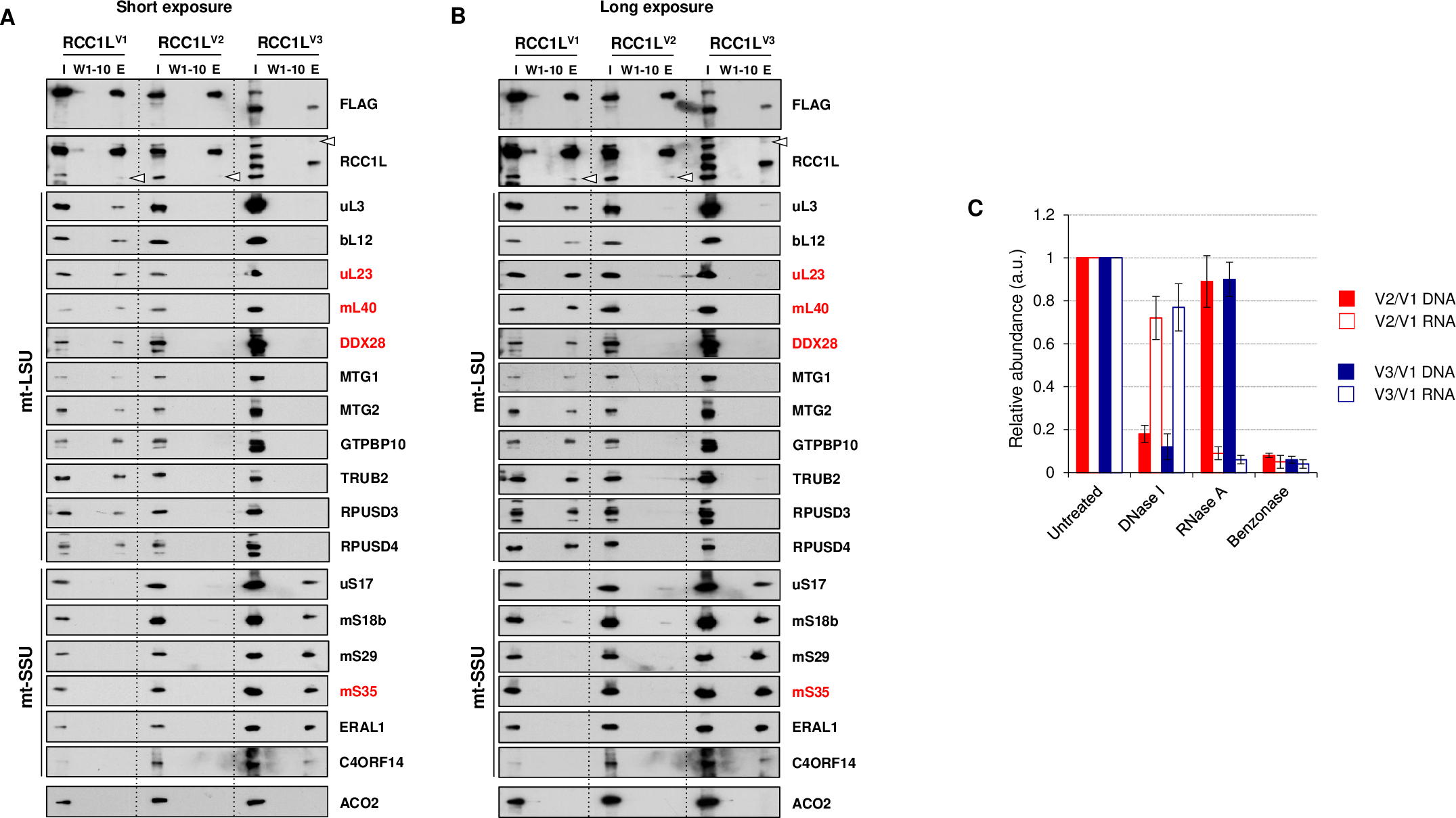

Supplement: S7 Fig — (A) Co-immunoprecipitation of STREP2-FLAG-tagged RCC1L isoforms from purified mitochondria after induction of HEK cells with 3–10 ng/ml doxycycline for 3–4 days. In the immunoblots of endogenous RCC1L, the endogenous isoforms (37 or 50 kDa band) are marked (empty arrowhead) in the elution fractions. Immunoblots for mitochondrial ribosomal proteins and biogenesis factors are presented. ACO2 was used as mitochondrial negative control for unspecific binding. Additional immunoblots not shown in Fig 4A are marked in red. (B) Longer exposure of the panel shown in (A). (C) Quantification of mitochondrial DNA and RNA by quantitative PCR (qPCR) in the elution fractions after DNAse I, RNAse A and Benzonase treatment. Data represent the average of MT-CO1, MT-CYB and 12S rRNA from two independent experiments and relative to the untreated samples. See S8 Table for quantitative data in this figure. (TIF) [file pgen.1008923.s007.tif]

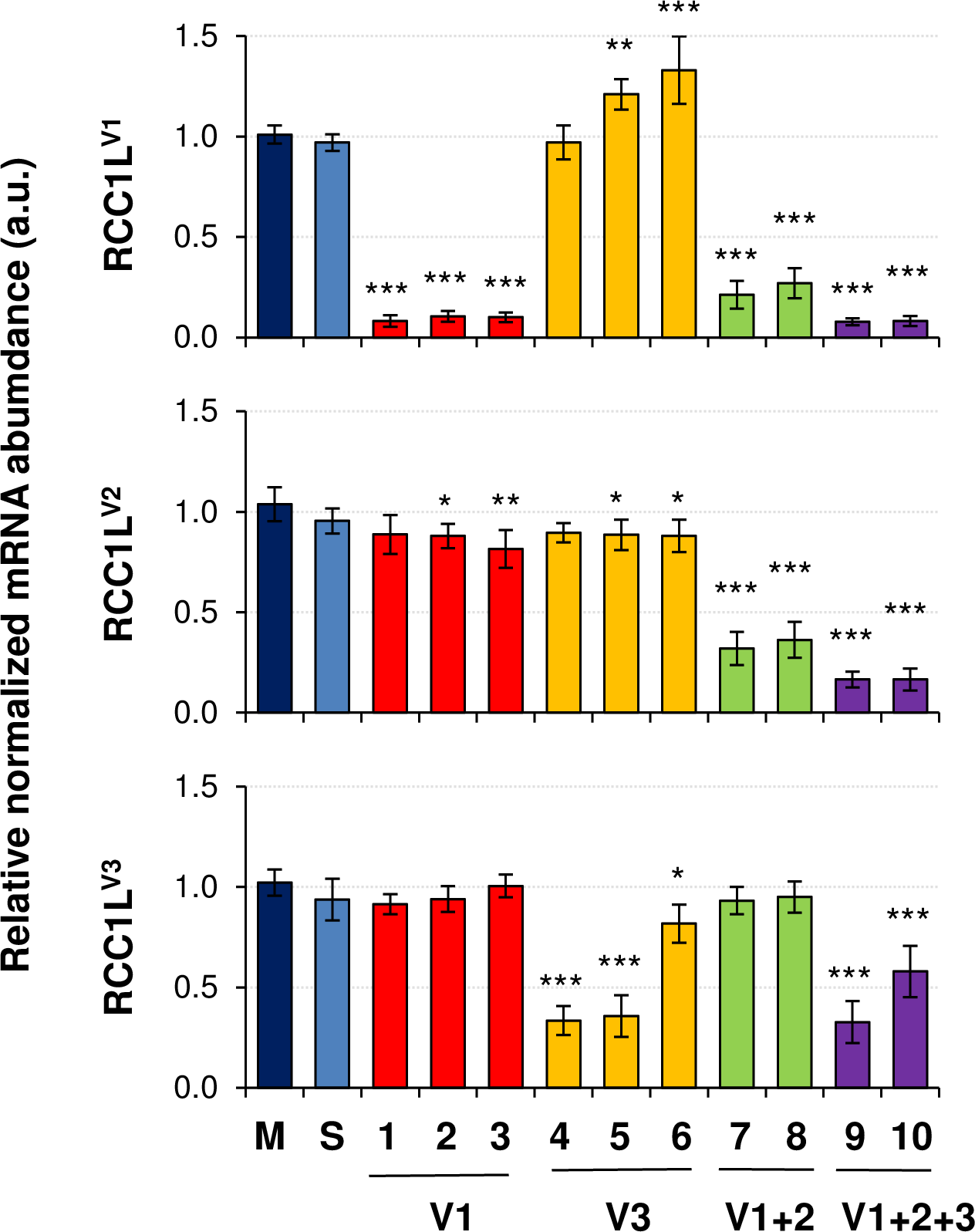

Supplement: S8 Fig — Quantification of isoform-specific mRNA levels by quantitative PCR (qPCR) in silencing experiments shown in Fig 6A and normalised to GAPDH. Silencing of RCC1LV1 (V1, RNAi 1–3) and RCC1LV3 (V3, RNAi 4–6) isoforms and combined silencing of RCC1LV1 and RCC1LV3 (V1+2, RNAi 7–8) and all three isoforms (V1+2+3, RNAi, 9–10) were performed in HEK cells. Mock (M) and AllStars dsRNA (S) transfected HEK cells were used as negative controls. Data represent mean ± SD from four independent experiments. t-test: *P < 0.05, **P < 0.01, ***P < 0.001. See S9 Table for quantitative data in this figure. (TIF) [file pgen.1008923.s008.tif]

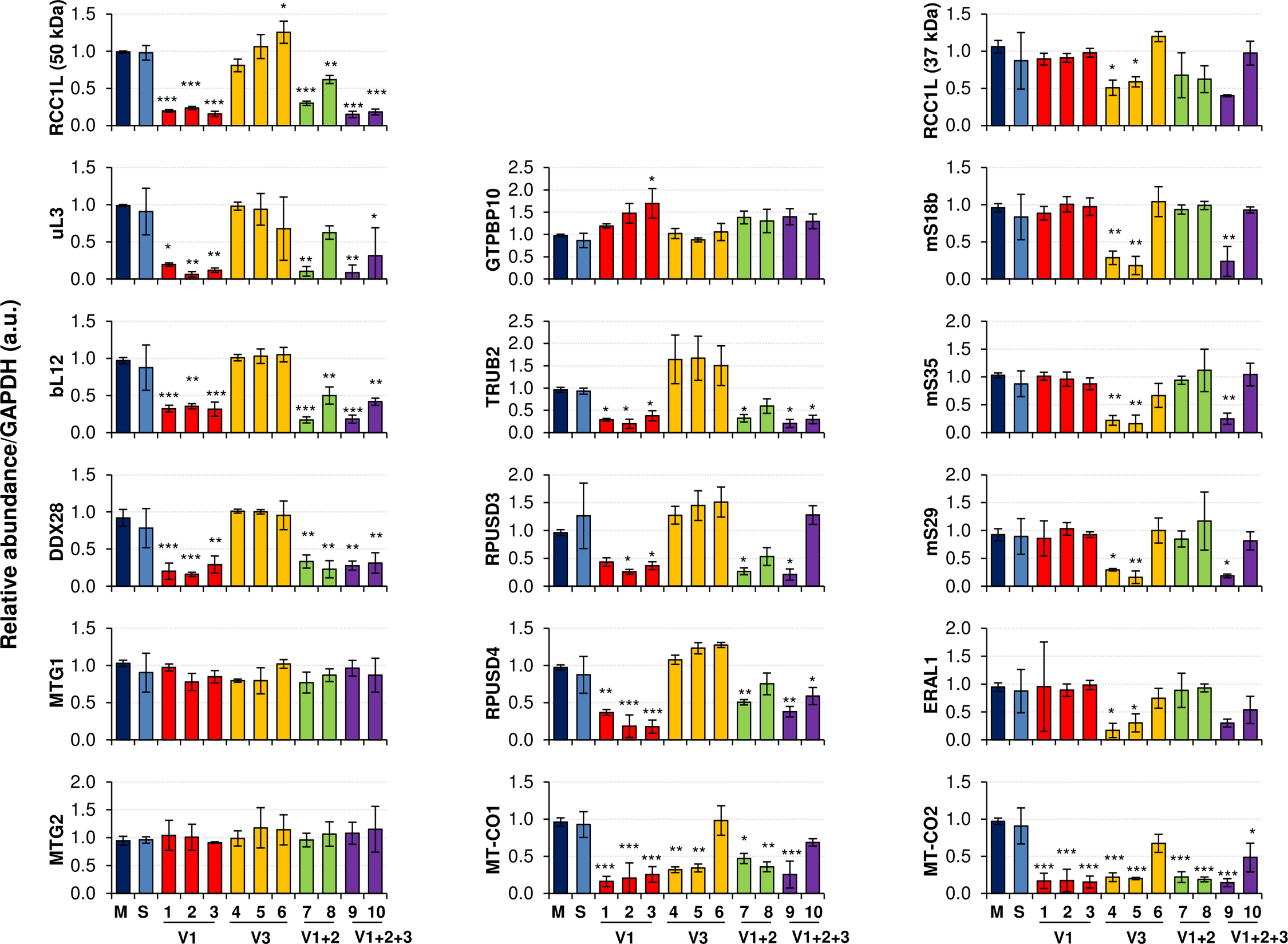

Supplement: S9 Fig — Quantification of protein levels in silencing experiments shown in Fig 6A and normalised to GAPDH. Silencing of RCC1LV1 (V1, RNAi 1–3) and RCC1LV3 (V3, RNAi 4–6) isoforms and combined silencing of RCC1LV1 and RCC1LV3 (V1+2, RNAi 7–8) and all three isoforms (V1+2+3, RNAi, 9–10) were performed in HEK cells. Mock (M) and AllStars dsRNA (S) transfected HEK cells were used as negative controls. Data represent mean ± SD from two independent experiments. t-test: *P < 0.05, **P < 0.01, ***P < 0.001. See S10 Table for quantitative data in this figure. (TIF) [file pgen.1008923.s009.tif]

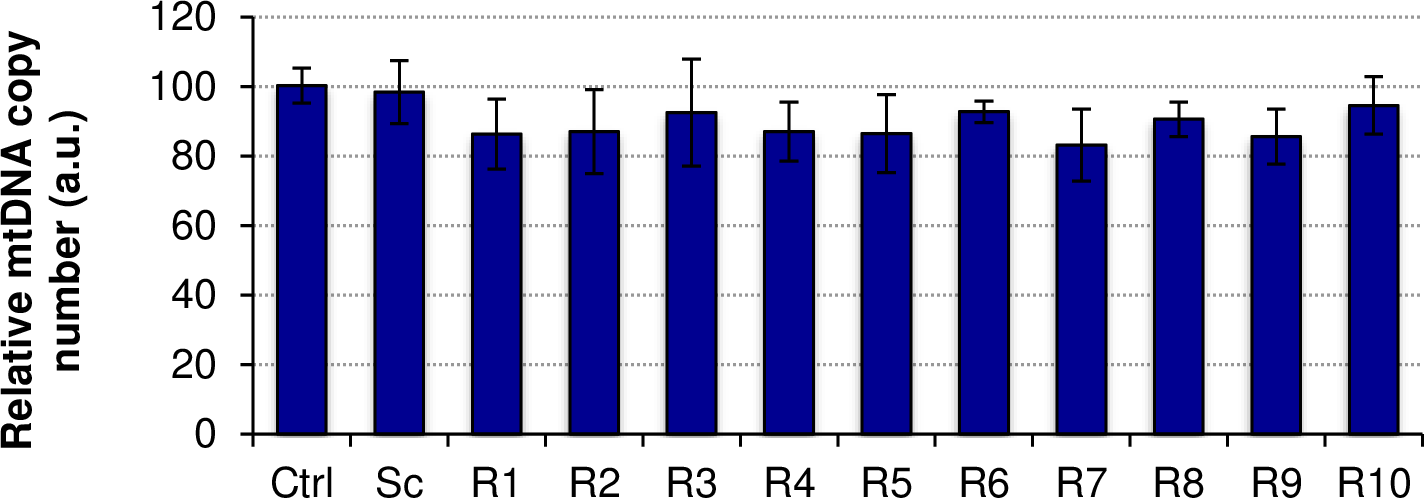

Supplement: S10 Fig — Quantitative PCR (qPCR) analysis of mitochondrial DNA copy number in silenced HEK cells. Silencing of RCC1LV1 (RNAi 1–3) and RCC1LV3 (RNAi 4–6) isoforms and combined silencing of RCC1LV1 and RCC1LV3 (RNAi 7–8) and all three isoforms (V1+2+3, RNAi, 9–10) in HEK cells. Mock (M) and AllStars dsRNA (S) transfected cells were used as negative controls. Data represent the average of MT-CO1, MT-CYB and 12S rRNA normalised to APP from three independent experiments. See S11 Table for quantitative data in this figure. (TIF) [file pgen.1008923.s010.tif]
